# Self-Assembling Lectin Nano-Block Oligomers Enhance Binding Avidity to Glycans

**DOI:** 10.3390/ijms23020676

**Published:** 2022-01-08

**Authors:** Shin Irumagawa, Keiko Hiemori, Sayoko Saito, Hiroaki Tateno, Ryoichi Arai

**Affiliations:** 1 Department of Biomolecular Innovation, Institute for Biomedical Sciences, Interdisciplinary Cluster for Cutting Edge Research, Shinshu University, Nagano 386-8567, Japan; 19hs103b@shinshu-u.ac.jp; 2 Department of Applied Biology, Faculty of Textile Science and Technology, Shinshu University, Nagano 386-8567, Japan; 3 Department of Science and Technology, Graduate School of Medicine, Science and Technology, Shinshu University, Nagano 386-8567, Japan; 4 Cellular and Molecular Biotechnology Research Institute, National Institute of Advanced Industrial Science and Technology (AIST), Ibaraki 305-8566, Japan; keiko-hiemori@aist.go.jp (K.H.); sayoko.saitou@aist.go.jp (S.S.); h-tateno@aist.go.jp (H.T.)

**Keywords:** artificial protein, avidity, fusion protein, lectin engineering, multivalent binding effect, protein complex design, protein nano-building block, protein oligomer

## Abstract

Lectins, carbohydrate-binding proteins, are attractive biomolecules for medical and biotechnological applications. Many lectins have multiple carbohydrate recognition domains (CRDs) and strongly bind to specific glycans through multivalent binding effect. In our previous study, protein nano-building blocks (PN-blocks) were developed to construct self-assembling supramolecular nanostructures by linking two oligomeric proteins. A PN-block, WA20-foldon, constructed by fusing a dimeric four-helix bundle de novo protein WA20 to a trimeric foldon domain of T4 phage fibritin, self-assembled into several types of polyhedral nanoarchitectures in multiples of 6-mer. Another PN-block, the extender PN-block (ePN-block), constructed by tandemly joining two copies of WA20, self-assembled into cyclized and extended chain-type nanostructures. This study developed novel functional protein nano-building blocks (lectin nano-blocks) by fusing WA20 to a dimeric lectin, *Agrocybe cylindracea* galectin (ACG). The lectin nano-blocks self-assembled into various oligomers in multiples of 2-mer (dimer, tetramer, hexamer, octamer, etc.). The mass fractions of each oligomer were changed by the length of the linkers between WA20 and ACG. The binding avidity of the lectin nano-block oligomers to glycans was significantly increased through multivalent effects compared with that of the original ACG dimer. Lectin nano-blocks with high avidity will be useful for various applications, such as specific cell labeling.

## 1. Introduction

Many proteins form oligomers and complexes, which perform advanced and cooperative functions, such as allosteric regulation and multivalent binding [1]. In protein engineering, the artificial design of proteins and oligomers/complexes is an attractive topic [2,3]. Many studies have reported constructing artificial protein oligomers and complexes using various approaches [4,5,6].

WA20 is a de novo protein obtained from a library of binary-patterned four-helix bundles [7,8]. The crystal structure of WA20 revealed an intermolecularly folded dimeric four-helix bundle with a bisecting U topology (PDB ID: 3VJF) [9]. Based on the dimeric structure of WA20, the stability of WA20 was improved by introducing five amino acid substitutions to enhance the hydrophobic core and α-helix stability [10]. This mutant (super WA20, SUWA) showed an extremely high denaturation midpoint temperature (*T*_m_) above the boiling point of water. Moreover, based on the rational prediction of stabilizing mutations using high-temperature molecular dynamics (MD) simulations, three mutations (N22A, N22E, and H86K) of WA20 were found. A double mutant (N22E and H86K) of SUWA (rationally optimized SUWA, ROSA) showed the highest *T*_m_ (129.0 °C) [11].

Furthermore, we developed protein nano-building blocks (PN-blocks) to construct self-assembling supramolecular nanostructures by utilizing the characteristic dimeric structure of WA20 as a component of artificial protein complexes. One of the PN-blocks, WA20-foldon, constructed by fusing the dimeric WA20 to a trimeric foldon domain of T4 phage fibritin, formed several types of self-assembled nanostructures in multiples of 6-mer including a barrel-like hexamer and a tetrahedrally shaped dodecamer [12]. In addition, we developed extender protein nano-building blocks (ePN-blocks) constructed by tandem joining two copies of WA20 with various linkers [13]. The ePN-blocks self-assembled into cyclized and extended chain-type nanostructures. Although various nanostructures have been constructed from PN-blocks, the development of functional PN-blocks remains a challenge.

Lectins are a class of proteins that bind to specific glycans through the carbohydrate recognition domain (CRD) [14,15]. Glycans cover various types of cells and generally exist as glycopolymers, as well as glycoconjugates appended to proteins and lipids. Changes in glycan composition and structure are related to many biological functions, such as interactions between cells, cancer progression, and microbial infections [16]; therefore, lectins have been studied in a wide range of fields of protein engineering [17,18,19] as candidates for medical applications, including as drug carriers for cancer therapy [20] and new antiviral drugs [21]. However, the affinity of lectins for glycans is lower than the affinity of the antibody for antigen [22,23], which is a major problem in the application of lectins. Many lectins have multiple CRDs within molecules through oligomerization or tandem repeats and enhance their avidity for glycans through multivalent binding effect [23,24]. Several studies have reported that the avidity of lectins can be enhanced by artificially increasing their multivalency through tandem repeats or oligomerization of CRDs [25,26,27].

*Agrocybe cylindracea* galectin (ACG) is a dimeric fungal lectin from the mushroom *A. cylindracea* [28] and it has a wide range of specificity for β-galactoside derivatives including Galβ1-3/4GlcNAc (LacNAc) and Galβ1-3GalNAc (T antigen). Especially, ACG preferentially bind the derivatives whose C-3 position of nonreducing terminal Gal is substituted with Siaα2-3, Sulfo-3, or GalNAcα1-3 [29,30,31]. ACG is a suitable target for lectin engineering [32] because its stable recombinant protein can be easily produced in *Escherichia* *coli.* Additionally, various ACG mutants that modify specificity have been reported [31,32,33,34,35].

To develop novel functional PN-blocks, we constructed lectin nano-building blocks (lectin nano-blocks) with the ability to bind to target glycans. The lectin nano-blocks, constructed by fusing WA20 to ACG with various linkers, formed several self-assembling oligomers in multiples of 2-mer (dimer, tetramer, hexamer, octamer, and decamer). The glycoconjugate microarray, hemagglutination assay, and cell staining experiments showed that the lectin nano-blocks had higher binding ability than recombinant ACG (rACG). Furthermore, we examined the binding ability of each oligomer of the lectin nano-blocks by surface plasmon resonance (SPR) experiments and found that larger oligomers tend to have higher binding ability. These results suggest that the construction of lectin nano-block oligomers is a useful strategy for enhancing the binding avidity of lectins.

## 2. Results

### 2.1. Design and Construction of Lectin Nano-Blocks

**Figure 1:**
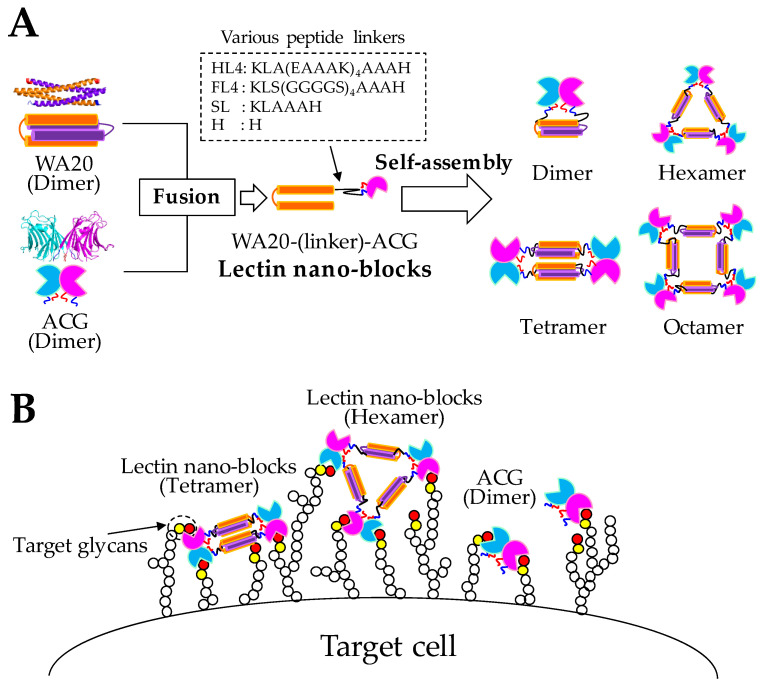
Schematics of the lectin nano-blocks. (**A**) Design of the lectin nano-blocks. The lectin nano-blocks were constructed by fusing the dimeric de novo protein WA20 (PDB ID: 3VJF) [9] to the dimeric lectin *Agrocybe cylindracea* galectin (ACG) (PDB ID: 1WW7) [30] with different type of linkers (HL4, FL4, SL, and H). In addition, WA20-ΔN3ACG was constructed by fusing WA20 and ACG without a linker and with the deletion of the N-terminal 3 aa of ACG. Since both WA20 and ACG form dimer, the lectin nano-blocks are expected to form self-assembling oligomers in multiples of 2-mer. (**B**) Schematics of the binding of the lectin nano-blocks and ACG to target glycans on cells. Because the lectin nano-block oligomers have more carbohydrate recognition domains (CRDs) than the original ACG, they are expected to enhance the binding avidity to target glycans by multivalent binding effect.

Lectin nano-blocks were designed by fusing the dimeric de novo protein WA20 to the dimeric lectin ACG (Figure 1). The WA20_H86K mutant was used as a component of the lectin nano-block in this study as it was a thermally stabilized mutant with a 3.5 °C higher denaturation midpoint temperature (*T*_m_) than the original WA20 [11] and formed a stable dimer in solution (Figure S1A). Thereafter, “WA20” refers to the WA20_H86K mutant in this study. The rACG also formed dimer in solution (Figure S1B). In constructing the lectin nano-blocks, we changed the length and rigidity of linkers between WA20 and ACG, which potentially affected the conformation of lectin nano-blocks. We designed lectin nano-blocks with various length and rigidity linkers [36] (Figure S2): WA20-HL4-ACG with helical linker 4 (HL4) consisting of 27 amino acids (aa) of KLA(EAAAK)_4_AAAH, which forms an α-helix [37,38]; WA20-FL4-ACG with flexible linker 4 (FL4) consisting of 27 aa of KLS(GGGGS)_4_AAAH, which is rich in glycine and serine residues; WA20-SL-ACG with a short linker (SL) consisting of 6 aa of KLAAAH; and WA20-H-ACG with H linker containing 1 aa of histidine, which is derived from the NdeI restriction site. WA20-ΔN3ACG is a lectin nano-block without a linker and with the deletion of the N-terminal 3 aa of ACG which is thought to have little effect on the dimeric structure and binding to glycans of ACG (Figure S3B). Because both WA20 and ACG usually form dimers (Figures S1 and S3), the lectin nano-blocks were expected to form various oligomers in multiples of 2-mer (Figure 1).

These five lectin nano-block proteins were expressed in *E. coli* and purified by immobilized metal affinity chromatography (IMAC) (Figure S4).

### 2.2. Structural Characterization of Lectin Nano-Blocks

#### 2.2.1. Size Exclusion Chromatography–Multi-Angle Light Scattering (SEC–MALS) Analysis

**Figure 2:**
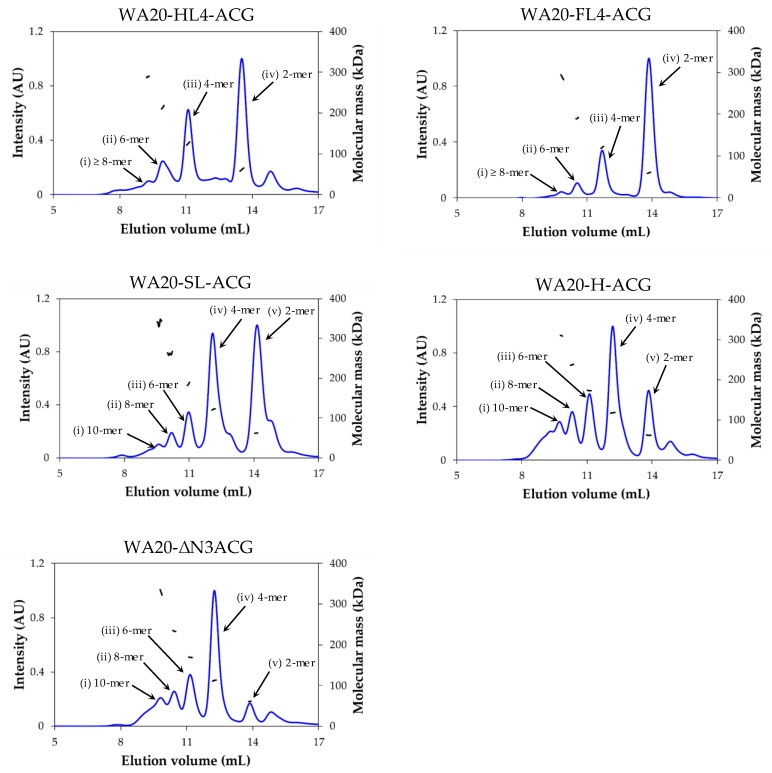
SEC–MALS profiles of the five lectin nano-blocks. The blue and the black lines represent the normalized intensity of UV absorbance (*A*_280 nm_) and the molecular mass of each peak, respectively.

To evaluate the oligomeric states of the five lectin nano-block proteins linked by different linkers, we performed SEC–MALS analysis on samples purified by IMAC. The chromatograms showed multiple UV peaks in all the lectin nano-block proteins (Figure 2), suggesting that they self-assembled into multiple oligomers, such as PN-blocks, as previously reported [12,13].

To estimate the oligomeric state of each peak, we calculated the values of the weight-average molecular mass (*M*_w_) divided by the theoretical molecular mass (*m*) of a monomer of each lectin nano-block (Table 1 and Figure S2). The values of each peak were close to multiples of two, regardless of the type of linker between WA20 and ACG. These results suggest that the lectin nano-blocks form regularly discrete oligomers in multiple 2-mer, such as dimer, tetramer, hexamer, octamer, and decamer, probably because of the combination of WA20 dimers and ACG dimers.

The mass fractions of each peak varied according to linker length (Table 1, Figure 2). The dimer peaks (iv) of WA20-HL4-ACG and WA20-FL4-ACG with long linkers occupied 47.3% and 66.8% of the total mass, respectively. The dimer peaks (v) of WA20-SL-ACG, WA20-H-ACG, and WA20-ΔN3ACG with short linkers or no linker occupied 39.2%, 16.5%, and 7.5% of the total mass, respectively. In contrast, the tetramer peaks (iii) of WA20-HL4-ACG and WA20-FL4-ACG with long linkers occupied 30.4% and 22.7% of the total mass, respectively. The tetramer peaks (iv) of WA20-SL-ACG, WA20-H-ACG, and WA20-ΔN3ACG with short linkers or no linker were 37.2%, 36.1%, and 44.8%, respectively. As the linkers became short, the mass fractions of the dimer peaks decreased and those of the tetramer and higher oligomer peaks increased. These results indicate that the linker length affects the formation of lectin nano-block oligomers.

#### 2.2.2. Small-Angle X-ray Scattering (SAXS) Analysis

**Figure 3:**
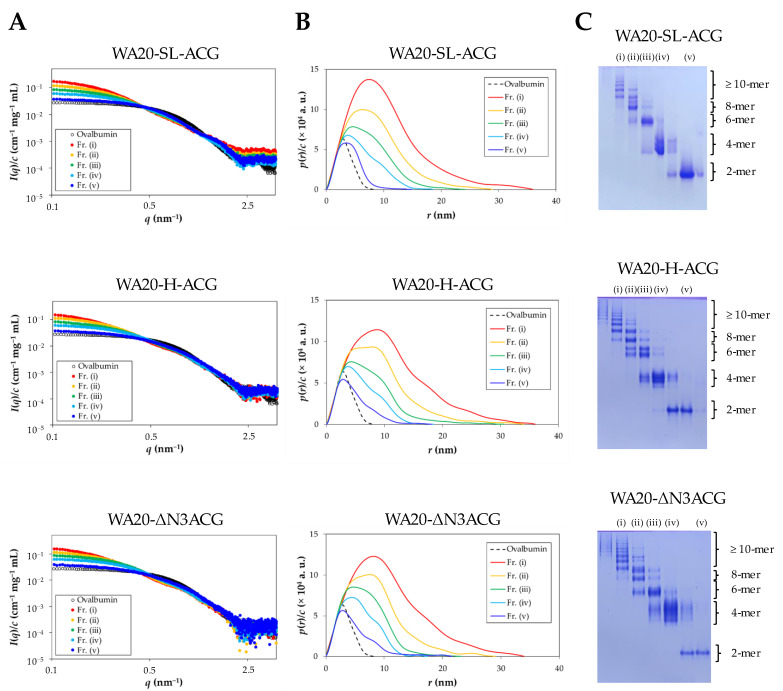
SAXS analysis. (**A**) Concentration-normalized absolute scattering intensities of the fractionated samples (Fr.) of WA20-SL-ACG, WA20-H-ACG, and WA20-ΔN3ACG. Ovalbumin was used as a reference standard of the molecular mass. (**B**) Concentration-normalized pair-distance distribution functions of WA20-SL-ACG, WA20-H-ACG, and WA20-ΔN3ACG obtained by inverse Fourier transforming the SAXS data. (**C**) Native PAGE analysis of the samples fractionated by SEC purification for the SAXS experiments. Proteins were stained with Coomassie brilliant blue. The oligomeric states of the protein bands were estimated from the SEC–MALS results.

To further analyze the lectin nano-block oligomers, small-angle X-ray scattering (SAXS) experiments were performed on WA20-SL-ACG, WA20-H-ACG, and WA20-ΔN3ACG fractionated by SEC purification (Figure S5). WA20-HL4-ACG and WA20-FL4-ACG mainly formed dimers, and the amount of higher oligomers required for SAXS experiments could not be prepared. Figure 3 and Figure S6 and Table 2 show a summary of the SAXS results and the weight-average molecular mass (*M*_w_) of each sample. Forward scattering intensity and radius of gyration (*R*_g_) were calculated from the Guinier plots (Figure S7 and Table 2). The *M*_w_ values of each fraction obtained from the SAXS experiments were approximately consistent with those obtained from the SEC–MALS analysis (Table 1). The pair-distance distribution functions (*p*(*r*)) indicate that higher oligomers of the lectin nano-blocks have larger maximum dimensions (*D*_max_) (Figure 3 and Figure S8 and Table 2).

**Figure 4:**
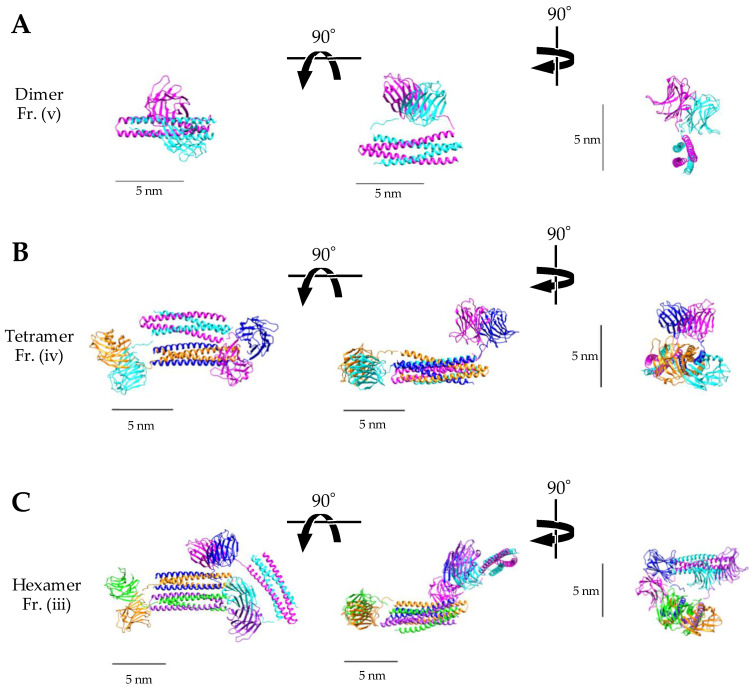
Rigid-body models of the lectin nano-block oligomers of WA20-SL-ACG. The rigid-body models of (**A**) dimer, (**B**) tetramer, and (**C**) hexamer of WA20-SL-ACG. The models were constructed based on the crystal structures of WA20 (PDB ID: 3VJF) [9] and ACG (PDB ID: 1WW7) [30] using the SAXS data and the rigid-body modelling program CORAL [39] without a symmetry constraint.

In addition, further analysis of the WA20-SL-ACG oligomers was performed to obtain more structural insights from the SAXS data by utilizing the high-resolution structures of WA20 and ACG since the dimer, tetramer, and hexamer of WA20-SL-ACG were purified mostly well in the result of native PAGE (Figure 3). The rigid-body models of each oligomer were constructed based on the crystal structures of the dimeric WA20 (PDB ID: 3VJF) [9] and the dimeric ACG (PDB ID: 1WW7) [30] using the CORAL (complexes with random loops) program [39]. The simulated SAXS intensity curves of all the constructed models were in agreement with the experimental data (Figure S9). As shown in Figure 4, the rigid-body models of the WA20-SL-ACG oligomers seem to be reasonable shapes that connect the WA20 and ACG structures.

### 2.3. Functional Characterization of Lectin Nano-Blocks

#### 2.3.1. Glycoconjugate Microarray Analysis

**Figure 5:**
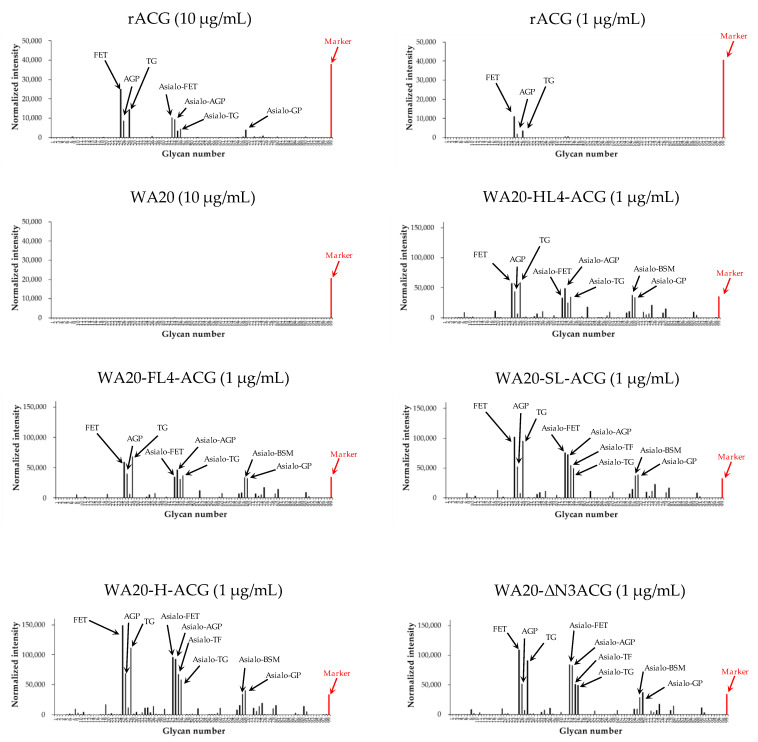
Glycoconjugate microarray analysis. Specificity profiles of rACG, WA20, and the five lectin nano-blocks. The Cy3-labeled proteins (1 µg/mL or 10 µg/mL) were applied to the array, and binding was detected by the scanner multiple times by varying the exposure time (0.1–3 s). The fluorescent intensity was normalized to the intensity per second. Abbreviations: FET, fetuin; AGP, α1-acid glycoprotein; TG, porcine thyroglobulin; TF, transferrin; BSM, bovine submaxillary mucin, and GP; human glycophorin.

To examine the binding specificity of the lectin nano-blocks, we performed a glycoconjugate microarray based on evanescent-field fluorescence-assisted detection [40] for the lectin nano-blocks, WA20, and rACG (Figure 5 and Figure S10). All the Cy3-labeled lectin nano-blocks and the rACG protein bound to almost the same glycans immobilized on the array, whereas WA20 did not bind to any glycans, suggesting that the lectin nano-blocks can bind to specific glycans through the ACG domain. The strongly bound glycans were several α2-3-sialylated glycoproteins including fetuin (FET), α1-acid glycoprotein (AGP), porcine thyroglobulin (TG), and several desialylated (asialo) glycoproteins (Table S1). These results were consistent with a wide range of specificity of wild-type ACG for β-galactoside derivatives in previous studies [29,30,31,33]. In addition, all the lectin nano-blocks showed higher fluorescence intensity than rACG at the same concentration (1 µg/mL). The samples of WA20-SL-ACG, WA20-H-ACG, and WA20-ΔN3ACG with a large amount of tetramer and higher oligomers tended to have higher fluorescence intensity than the samples of WA20-HL4-ACG and WA20-FL4-ACG.

#### 2.3.2. Hemagglutinating Activity of Lectin Nano-Blocks

To examine the biological activity of the lectin nano-blocks, we performed a hemagglutination assay against rabbit erythrocytes (Figure S11). All the lectin nano-blocks and rACG agglutinated erythrocytes; however, WA20 did not agglutinate erythrocytes. All the lectin nano-blocks had a smaller minimum concentration for agglutination (MCA) than rACG (Table 3), indicating that the lectin nano-blocks exhibited stronger hemagglutinating activity than rACG.

#### 2.3.3. Cell Staining Experiments with Lectin Nano-Blocks

**Figure 6:**
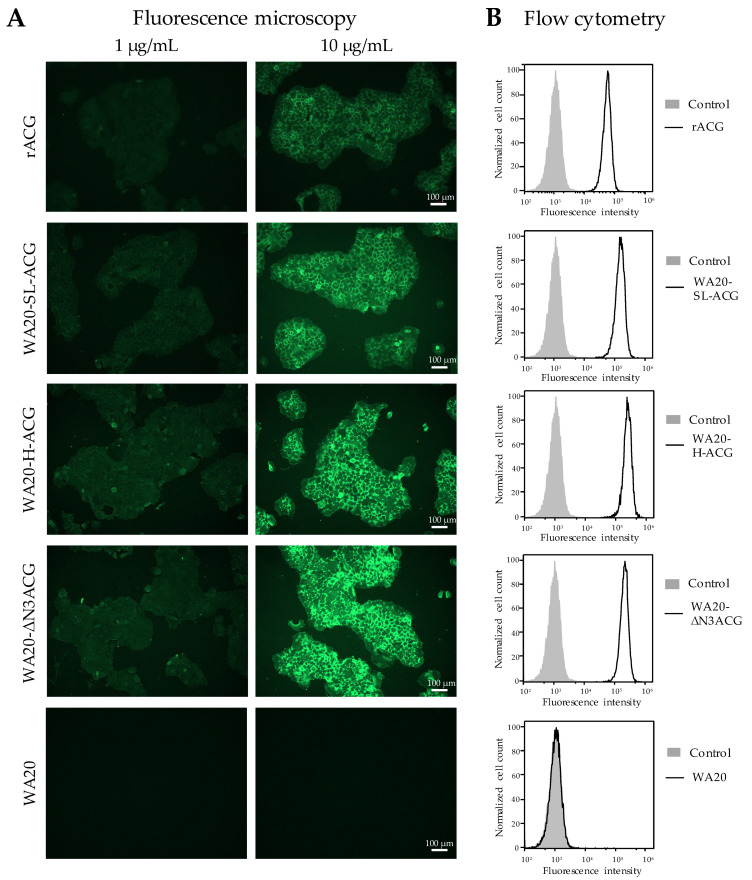
Cell staining experiments. (**A**) Fluorescence microscopy images. BxPC-3 cells of a human pancreatic cancer cell line were stained with the fluorescein-labelled samples of lectin nano-blocks, rACG, and WA20. (**B**) Flow cytometry results. The experiments were performed on BxPC-3 cells. The histogram of each sample is shown with a black line. The negative control, fluorescein isothiocyanate (FITC)-labelled bovine serum albumin (BSA), is shown in the gray area.

To examine whether the lectin nano-blocks could be of practical use for detecting target cells with specific glycans, we performed cell staining experiments on the lectin nano-blocks (Figure 6), WA20-SL-ACG, WA20-H-ACG, and WA20-ΔN3ACG, which tend to bind to specific glycans more strongly than do WA20-HL4-ACG and WA20-FL4-ACG, according to the results of the glycoconjugate microarray (Figure 5). The lectin nano-blocks (WA20-SL-ACG, WA20-H-ACG, and WA20-ΔN3ACG) and rACG, labeled with fluorescein, stained the cells of the human pancreatic cancer cell line BxPC-3, presenting some sialyl glycoepitopes [41], whereas fluorescein-labeled WA20 did not stain the cells (Figure 6A). The cells stained with the lectin nano-blocks appeared brighter than the cells stained with rACG, suggesting that the lectin nano-blocks bound to the target cells more strongly than rACG.

Moreover, flow cytometry analysis of the stained cells showed a higher binding ability of the lectin nano-blocks to BxPC-3 cells than that of rACG (Figure 6B).

#### 2.3.4. Surface Plasmon Resonance (SPR) Analysis of Lectin Nano-Block Oligomers

To examine multivalent binding effect, we performed SPR analysis for the lectin nano-block oligomers fractionated by SEC purification (Figure S12). We focused on WA20-H-ACG and WA20-ΔN3ACG for SPR experiments as they contained a relatively high proportion of large oligomers (tetramers, hexamers, octamers, and decamers) (Table 1).

**Figure 7:**
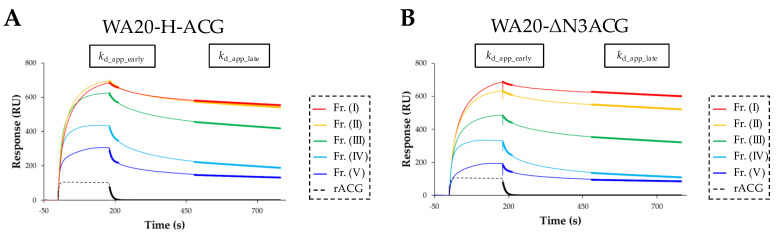
SPR analysis. Sensorgrams of the fractionated samples (Fr.) of the lectin nano-block oligomers of (**A**) WA20-H-ACG and (**B**) WA20-ΔN3ACG at 1 µM bound to the 3′-sialyllactose ligand. The sensorgram of rACG at 1 µM is also shown for comparison. Sensorgrams of all samples at all concentrations are shown in Figures S13 and S14. Bold lines show the fitting curves used for calculating the apparent dissociation rate constants *k*_d_app_early_ in the early phase (181–211 s) and *k*_d_app_late_ in the late phase (480–780 s) of the lectin nano-block oligomers. The apparent dissociation rate constant *k*_d_app_ of rACG was calculated using the data in the entire dissociation time (181–780 s).

The sensorgrams showed that rACG and all oligomers of WA20-H-ACG and WA20-ΔN3ACG bound to 3′-sialyllactose polyacrylamide biotin conjugate (Neu5Acα2-3Galβ1-4Glcβ-Gly-PAA-biotin) ligand immobilized on a streptavidin sensor chip, whereas WA20 did not bind to the ligand (Figure 7, Figures S13 and S14). The sensorgram of rACG showed a box-shaped response, which is typical for rapid association and dissociation. The sensorgrams of all the oligomers of the lectin nano-blocks showed higher responses with a slow dissociation. The sensorgrams of the lectin nano-block oligomers containing larger oligomers showed higher response values (Figure 7), suggesting that the larger multivalent oligomers of the lectin nano-blocks have higher binding avidity to the glycans.

Because it is practically difficult to apply any available binding models of kinetic analysis to the SPR data of the lectin nano-blocks due to multivalent binding, we attempted to roughly estimate the apparent dissociation constant (*K*_D_app_) by applying a 1:1 binding steady state model (Table 4, Figures S15 and S16). The *K*_D_app_ of each lectin nano-block oligomer (~10^−6^–10^−7^ M) was smaller than that of rACG (2.0 × 10^−5^ M), suggesting that the lectin nano-blocks have a higher binding ability than rACG. The larger oligomers tended to have a smaller *K*_D_app_, suggesting that the binding avidity is increased by the multivalent binding effect.

Although it is practically impossible to analyze association rate constants due to multivalent binding in the lectin nano-blocks, to roughly estimate apparent dissociation rate constants, the SPR data in the dissociation phase were fitted to the pseudo-first-order kinetic equation (Figure 7, Figures S13 and S14). The response of rACG rapidly returned to the baseline in the dissociation phase, and the apparent dissociation rate constant (*k*_d_app_) was approximately calculated using the entire dissociation data from 181 to 780 s. In contrast, the sensorgrams of the lectin nano-blocks in the dissociation phase showed fast dissociation in the early phase and very slow dissociation in the late phase; their response did not return to the baseline within the measurement time, suggesting that the dissociation data of the lectin nano-blocks consist of different components of dissociation rates. We hypothesized that the fast dissociation rate component dominantly appears in the early dissociation phase (181–211 s), and the slow dissociation rate component dominantly appears in the late dissociation phase (480–780 s). The dissociation data of the early phase (181–211 s) and the late phase (480–780 s) were separately fitted to the pseudo-first-order kinetic equation [42] to calculate the different components of the apparent dissociation rate constants in the early phase (*k*_d_app_early_) and the late phase (*k*_d_app_late_) (Table 4). Compared with the *k*_d_app_ of rACG, the *k*_d_app_early_ of the lectin nano-block oligomers was not very different, whereas the *k*_d_app_late_ of the lectin nano-block oligomers was much smaller than that of rACG. These results suggest that the improvement in the binding ability of the lectin nano-blocks may be attributed in part to the very slow dissociation rate component found in the data of the lectin nano-block oligomers.

## 3. Discussion

In many lectins, binding avidity to target glycans is enhanced by forming multivalent oligomers [23,24]. To enhance the avidity of ACG, this study developed the lectin nano-blocks by linking the dimeric artificial protein WA20 to the dimeric lectin ACG (Figure 1). The SEC–MALS results showed that all lectin nano-blocks with various linkers form oligomers in multiples of 2-mer probably because of the combination of WA20 dimers and ACG dimers (Figure 2 and Table 1). In addition, according to the native PAGE results (Figure S17), the SEC fractionated oligomers of the lectin nano-blocks did not change essentially to the other oligomeric states for five weeks at 4 °C, suggesting that each lectin nano-block oligomer is very stable and does not exchange with other oligomeric states on a timescale of several weeks.

The formation of each lectin nano-block oligomer was affected by the linker length between WA20 and ACG. The mass fractions of tetramer and higher oligomers increased in the lectin nano-blocks with short linkers (WA20-SL-ACG and WA20-H-ACG) and without a linker (WA20-ΔN3ACG), whereas the lectin nano-blocks with long linkers (WA20-FL4-ACG and WA20-HL4-ACG) preferentially formed dimers. The distance between the C-termini of the WA20 dimer (PDB ID: 3VJF) [9] is ~6 nm and the distance between the N-termini of the ACG dimer (PDB ID: 1WW7) [30] is ~2 nm (Figure S18A). Because there is a gap between the distance of the C-termini of the WA20 dimer and the distance of the N-termini of the ACG dimer, the lectin nano-blocks cannot form the usual dimers of WA20 and ACG simultaneously when the WA20 domain and the ACG domain are connected with an H linker that is too short or without a linker (ΔN3), as shown in Figure S18B. Thus, the lectin nano-blocks of WA20-H-ACG and WA20-ΔN3ACG preferentially formed tetramers and higher oligomers (Figure 2 and Table 1). In contrast, when the WA20 and ACG domains are connected with long linkers (FL4, HL4), the linkers are long enough to form the usual dimers of WA20 and ACG (Figure S18B), and the lectin nano-blocks of WA20-FL4-ACG and WA20-HL4-ACG preferentially formed dimers (Figure 2 and Table 1).

In addition, SAXS analysis of the fractionated oligomers of WA20-SL-ACG, WA20-H-ACG, and WA20-ΔN3ACG provided the structural information (Figure 3 and Table 2). As shown in Figure S8A, the shape of the *p*(*r*) function of the WA20-SL-ACG dimer was different from those of the WA20-H-ACG dimer and the WA20-ΔN3ACG dimer. To obtain structural insights into these dimers, low-resolution dummy atom models were constructed based on the SAXS data (Figure S19). The ab initio dummy atom models suggest that the WA20-SL-ACG dimer had a shape corresponding to the combination of the crystal structures of WA20 and ACG, whereas the models of the WA20-H-ACG dimer and the WA20-ΔN3ACG dimer had more elongated shapes than those of the WA20-SL-ACG dimer. This suggests that the structures of the WA20-H-ACG dimer and the WA20-ΔN3ACG dimer may be deformed from the original crystal structures of WA20 and ACG, as shown in Figure S18B.

In the case of the rigid-body model of the WA20-SL-ACG dimer (Figure 4A), the SL linker seems to have almost the minimum distance to form the usual dimers of WA20 and ACG, suggesting that the SL linker may slightly affect the formation of the dimer and consequently the dimer and the tetramer of WA20-SL-ACG formed in approximately the same amounts (Figure 2 and Table 1). In contrast, the rigid-body models of the WA20-H-ACG dimer and the WA20-ΔN3ACG dimer could not be constructed because of the too short linker or no linker connecting the WA20 and ACG structures.

The experimental results of the glycoconjugate microarray analysis (Figure 5), hemagglutination assay (Table 3), and the cell staining experiments (Figure 6) show that the lectin nano-blocks have the same specificity as rACG and the higher binding ability for the target glycans than rACG, as intended. To examine whether these functional improvements are attributed to multivalent binding effect, we performed SPR analysis of the fractionated samples of the WA20-H-ACG and WA20-ΔN3ACG oligomers (Figure 7, Figures S13 and S14). In the SPR results of the lectin nano-blocks, the larger oligomers showed relatively higher *R*_max_app_, smaller *K*_D_app_, smaller *k*_d_app_early_, and smaller *k*_d_app_late_, suggesting an improvement in their avidity through the enhanced multivalent binding effect (Table 4). However, the dimers of WA20-H-ACG and WA20-ΔN3ACG, which have two binding sites, as in the case of rACG dimer, also showed significant improvements in the binding ability to glycans compared with rACG (Table 4), suggesting a factor enhancing the binding ability of the lectin nano-blocks other than the multivalent binding effect. In this experiment, Neu5Acα2-3Galβ1-4Glcβ-Gly-PAA-biotin, a polyacrylamide biotin conjugate with many target glycans (3′-sialyllactose), was used as a ligand to examine the multivalent binding effect. Binding of biotin to tetrameric streptavidin was used for immobilization. Multivalent interactions with many target glycans densely conjugated with polyacrylamide chains immobilized on the sensor chip may slow the dissociation of the lectin nano-block oligomers with a large molecular size and enhance their reassociation, possibly resulting in the very slow dissociation rate component of the lectin nano-block oligomers.

In this study, we developed lectin nano-blocks by fusing the dimeric de novo protein WA20 and the dimeric lectin ACG, and the lectin nano-block oligomers obtained higher avidity to the target glycans than rACG through the multivalent binding effect. Because the lectin nano-block strategy demonstrated in this study can be applied to a variety of oligomeric lectins, this strategy is a useful method to improve the avidity of lectins, contributing to lectin engineering and applications.

## 4. Materials and Methods

### 4.1. Construction of WA20-ACG Protein Expression Plasmids

The protein expression plasmids of the lectin nano-blocks of WA20-HL4-ACG, WA20-FL4-ACG, and WA20-SL-ACG were constructed using the plasmids pET_WA20-HL4-WA20 [13], pET_WA20-FL4-WA20 [13], and pET_WA20-(SL)-foldon [12]. The H86K mutation of WA20 was introduced by site-directed mutagenesis of the transfer-PCR method [43] with oligo-DNA primers (Table S2) and KOD-Plus-Neo DNA polymerase (Toyobo, Osaka, Japan). The DNA fragment encoding ACG was prepared by digestion of the pET27b_rACG plasmid [31] with NdeI and XhoI, and cloned into the pET plasmids with the genes of WA20_H86K and each linker to give the expression plasmids pET_WA20-HL4-ACG (Figure S20A), pET_WA20-FL4-ACG, and pET_WA20-SL-ACG. The protein expression plasmids of WA20-H-ACG and WA20-ΔN3ACG were prepared from pET_WA20-SL-ACG using inverse PCR with the oligo-DNA primers (Table S2) and DNA ligation to give the expression plasmids pET_WA20-H-ACG and pET_WA20-ΔN3ACG (Figure S20B).

### 4.2. Protein Expression and Purification

The five lectin nano-blocks were expressed in *E. coli* BL21 Star (DE3) (Invitrogen, Carlsbad, CA, USA) harboring the expression plasmid in 2 L of LB broth (Lennox) (Nacalai Tesque, Kyoto, Japan) containing 100 µg/mL ampicillin sodium salt at 37 °C. The expression was induced by 0.1 mM isopropyl β-_D_-1-thiogalactopyranoside (IPTG) at an optical density of 600 nm (OD_600_) of ~0.6 and cells were cultured for 16 h at 16 °C. Proteins were extracted from the harvested cells by freezing–thawing and sonication with a VC 505 ultrasonic processor (Sonics and Materials, Newtown, CT, USA) in 50 mM sodium phosphate buffer (pH 7.5) containing 300 mM NaCl and 10% glycerol. The proteins were purified by immobilized metal affinity chromatography (IMAC) with TALON metal affinity resin (Takara Bio, Kusatsu, Shiga, Japan). Because many histidine residues are exposed on the surface of WA20 (PDB ID: 3VJF), the lectin nano-blocks without a His-tag can bind to the IMAC resin. These resins were washed with 50 mM sodium phosphate buffer (pH 7.5) containing 300 mM NaCl and 10% glycerol, and the proteins were eluted with 50 mM sodium phosphate buffer (pH 7.5) containing 300 mM NaCl, 10% glycerol and 200 mM imidazole. The protein concentration was determined by measuring the absorbance at 280 nm using a NanoDrop Lite spectrophotometer (Thermo Fisher Scientific, Waltham, MA, USA). The molar extinction coefficient of each protein was calculated according to the amino acid sequence (Trp: 5500, Tyr: 1490) [44]. The WA20_H86K protein, called as WA20 in this study, was expressed in *E. coli* BL21 Star (DE3) harboring the expression plasmid pET_WA20_H86K in 1 L LB broth (Lennox), as described in a previous study [11]. *E. coli* cells were cultured for 16 h without induction by IPTG. The protein was extracted from the harvested cells and purified by IMAC in the same way as the lectin nano-blocks.

The rACG protein was also expressed in *E. coli* BL21 Star (DE3) harboring the expression plasmid pET27b_rACG in 1 L of LB broth (Lennox) containing 30 µg/mL kanamycin sulfate at 37 °C. The expression was induced with 0.1 mM IPTG at OD_600_ = ~0.6, and cells were cultured for 16 h at 20 °C. The rACG protein was extracted from the harvested cells by freezing–thawing and sonication with a VC 505 ultrasonic processor (Sonics and Materials, Newtown, CT, USA) in 50 mM sodium phosphate buffer (pH 7.5) containing 300 mM NaCl and 10% glycerol. The rACG protein was purified by affinity chromatography with lactose-immobilized Sepharose CL-4B (Cytiva, Little Chalfont, Buckinghamshire, U.K.) [45]. The resin was washed with 50 mM sodium phosphate buffer (pH 7.5) containing 300 mM NaCl and 10% glycerol, and rACG was eluted with 50 mM sodium phosphate buffer (pH 7.5) containing 300 mM NaCl, 10% glycerol, and 200 mM lactose.

Before the glycoconjugate microarray, hemagglutination assay, and cell staining experiments, the protein samples were dialyzed overnight against 20 mM HEPES buffer (pH 7.5) containing 150 mM NaCl.

### 4.3. Size Exclusion Chromatography–Multi Angle Light Scattering (SEC–MALS)

The SEC–MALS experiments for samples purified by IMAC were performed using an Alliance e2695 HPLC system (Waters, Milford, MA, USA) equipped with a Superdex 200 Increase 10/300 GL column (Cytiva), which was connected in line with a DAWN HELEOS II multi-angle static light scattering detector (Wyatt Technology, Santa Barbara, CA, USA). The data were collected at 20 °C with 20 mM HEPES buffer (pH 7.5) containing 150 mM NaCl and analyzed using ASTRA 6 software (Wyatt Technology) [46]. Protein concentration was determined using a refractive index detector. A d*n*/d*c* value of 0.185 mL/g was generally used for the proteins.

### 4.4. Small-Angle X-ray Scattering (SAXS)

For the SAXS experiments, the protein samples were further purified by SEC (20 mM HEPES buffer (pH 7.5) containing 150 mM NaCl and 5% glycerol) with a Superdex 200 Increase 10/300 GL column (Cytiva). SAXS measurements were performed for samples (~1–4 mg/mL) and chicken ovalbumin (5 mg/mL) (A7641; Sigma-Aldrich, St. Louis, MO, USA) dissolved in the HEPES buffer at 20 °C using synchrotron radiation (λ = 1.0 Å) at the Photon Factory (PF) BL-10C beamline, High Energy Accelerator Research Organization (KEK) (Tsukuba, Japan) [47] with a PILATUS3 2M detector (Dectris, Baden, Switzerland) at a sample-detector distance of 2.08 m.

The two-dimensional scattering images were radially integrated into one-dimensional scattering intensities *I*(*q*) as a function of the magnitude of the scattering vector, as shown by the following equation using SAngler [48]:

*q* = (4π/λ) sin(*θ*/2) (1)

In this equation, *θ* means the total scattering angle.

The indirect Fourier transformation (IFT) technique was used to calculate the pair-distance distribution function *p*(*r*) using GNOM [49] in the ATSAS program suite [50]. The forward scattering intensity, *I*(*q*→0), and radius of gyration, *R*_g_, were estimated using the Guinier approximation [51] in the range of *qR*_g_ < 1.3 using the ATSAS program [50] with SAngler [48]. Assuming that the proteins have practically the same scattering length density and specific volume and that the structure factor is almost unity (*S*(*q*) ≈ 1) for the dilute samples, the forward scattering intensity normalized by the protein concentration (mg/mL), *I*(*q*→0)/*c*, is proportional to the weight average molecular mass (*M*_w_). Ovalbumin (*M*_w_ = 44.3 kDa) was used as the reference standard for the molecular mass.

The low-resolution dummy atom models were constructed from the SAXS data using ab initio shape modelling programs in the ATSAS program suite [50] for small-angle scattering data analysis from biological macromolecules. The calculations of rapid ab initio shape determination were performed ten times by DAMMIF [52] without a symmetry constraint, and the generated models were aligned and averaged using DAMAVER [53]. The averaged model was modified with the fixed core by DAMSTART, and further refinement of the model was performed by DAMMIN [54]. The images of the dummy atom models were prepared using UCSF Chimera [55].

The rigid-body models of the WA20-SL-ACG oligomers were constructed from the SAXS data based on the crystal structures of WA20 (PDB ID: 3VJF) [9] and ACG (PDB ID: 1WW7) [30] using CORAL [39] in the ATSAS program suite [50]. For the construction of the models, the residue R102 was disordered in the crystal structure at the C-terminus of WA20, the SL linker residues (six residues) between WA20 and ACG, and the three residues at the N-terminus of ACG were regarded as random loop residues because the three residues at the N-terminus in the crystal structure of ACG seem to have a relatively flexible structure. The images of the rigid-body models were prepared using UCSF Chimera [55].

### 4.5. Glycoconjugate Microarray

The general experimental procedure was described in a previous study [40]. Proteins were fluorescently labeled with Cy3 mono-reactive dye (Cytiva). Thereafter, 80 µL of Cy3-labeled proteins (at a final concentration of 1.0 µg/mL or 10 µg/mL) was applied to the glycoconjugate microarray and incubated overnight at 20 °C. After the wells on the glass were rinsed twice with the probing buffer (Tris-buffered saline (pH 7.4) containing 1% Triton X-100, 1 mM CaCl_2_, and 1 mM MnCl_2_), fluorescence images were acquired using an evanescent-field activated fluorescence scanner (Bio-REX Scan 200; Rexxam, Osaka, Japan). The net intensity value of each spot was obtained by subtracting the background from the signal intensity. The signals of the three spot samples were averaged, and the intensities were normalized by the exposure time.

### 4.6. Hemagglutination Assay

Serial 2-fold dilutions of 25 µL of the protein samples (initial concentration: 1 µM) in HEPES buffer (20 mM HEPES buffer (pH7.5), 150 mM NaCl) were prepared in a U-bottom 96-well microtiter plate. Thereafter, 50 µL of 2% glutaraldehyde-stabilized rabbit red blood cells (Cedarlane, Burlington, ON, Canada) in HEPES buffer was added to each well in the plate, and the plate was shaken gently (150 rpm) for 30 min at 20 °C. The plate was then statically incubated for 30 min at 20 °C. The threshold concentration of the protein sample that caused hemagglutination was determined as the minimum concentration for agglutination (MCA).

### 4.7. Cell Staining Experiment

The protein samples of rACG, WA20-SL-ACG, WA20-H-ACG, WA20-ΔN3ACG, and WA20 were fluorescently labeled using Fluorescein Labeling Kit-NH_2_ (Dojindo, Kumamoto, Japan). After cells of the human pancreatic cancer cell line BxPC-3 were cultured in 24-well plates, the cells were washed with phosphate buffered saline (PBS) and fixed in 4% paraformaldehyde for 20 min. After washing the cells with PBS three times, the cells were incubated with 1 µg/mL or 10 µg/mL of the fluorescein-labeled protein samples for 1 h at room temperature. After washing twice with PBS, the cells were observed under a fluorescence microscope.

### 4.8. Flow Cytometry

BxPC-3 cells (1 × 10^5^) were washed with 1% BSA in PBS and incubated with 10 µg/mL of the fluorescein-labeled protein samples or FITC-labeled BSA on ice for 1 h. After two cycles of centrifugation and washing with 1% BSA in PBS, the cells were analyzed using a CytoFLEX flow cytometer (Beckman Coulter, Brea, CA, USA).

### 4.9. Surface Plasmon Resonance (SPR) Analysis

For the SPR experiments, the lectin nano-blocks (WA20-H-ACG and WA20-ΔN3ACG) and rACG were further purified by SEC (20 mM HEPES buffer (pH 7.5) containing 150 mM NaCl) with a Superdex 200 Increase 10/300 GL column (Cytiva). The IMAC-purified WA20 was dialyzed overnight against 20 mM HEPES buffer (pH 7.5) containing 150 mM NaCl. Molar concentrations of protein samples were calculated as monomers. SPR measurements were performed at 25 °C using a Biacore T200 (Cytiva) equipped with a Series S Sensor Chip SA (Cytiva), whose surface consisted of a carboxymethylated dextran matrix pre-immobilized with streptavidin, and 20 mM HEPES buffer (pH 7.5) containing 150 mM NaCl and 0.05% Tween 20 was used as the running buffer. Neu5Acα2-3Galβ1-4Glcβ-Gly-PAA-biotin ligand (GlycoNZ, Auckland, New Zealand) was immobilized on streptavidin on the chip surface (RU = 82.1). The response of each protein sample was measured at a flow rate of 30 µL/min, binding time of 180 s, and dissociation time of 600 s. The amount of binding of each protein sample to the ligand was determined by subtracting the response of the analytical flow cell to that of the reference flow cell that was not immobilized as the background. The sensor chip was regenerated by flowing 20 mM HEPES buffer (pH 7.5) containing 150 mM NaCl, 0.05% Tween20, and 200 mM lactose at 30 µL/min for 120 s after each measurement.

The calculation of the apparent amount of maximum binding (*R*_max_app_) and apparent dissociation constant (*K*_D_app_) were calculated by fitting the data of multiple protein concentrations of each sample to the 1:1 binding steady-state affinity model in BIAevaluation software (version 3.0). In this model, *K*_D_app_ is calculated by regarding it as an apparent first-order binding reaction between the analyte and ligand, as shown in the following equation:

*R*_eq_ = *c R*_max_app_/(*K*_D_app_ + *c*) + offset (2)

In this equation, *c* is the concentration of the analyte and *R*_eq_ is the response at the equilibrium state for each measured concentration.

The apparent dissociation rate constants in the early dissociation phase (181–211 s) (*k*_d_app_early_) and the late dissociation phase (480–780 s) (*k*_d_app_late_) were calculated by globally fitting the SPR data of several protein concentrations to the following equation for the classical pseudo-first-order kinetics [42] using Igor Pro (version 7.08) (WaveMetrics, Portland, OR, USA) with global fitting analysis package.

For the *k*_d_app_early_ in the early dissociation phase (181–211 s):

*R*_t_ = *R*_0_ exp(− *k*_d___app_early_ (*t* − *t*_0_)) + *R*_1_ (3)

For the *k*_d_app_late_ in the late dissociation phase (480–780 s):

*R*_t_ = *R*_0_ exp(− *k*_d_app_late_ (*t* − *t*_0_)) (4)

In this equation, *R*_t_ is the response at time *t*, *R*_0_ is the response strength at *t*_0_ (*k*_d_app_early_: 181 s, *k*_d_app_late_: 480 s, respectively), and *R*_1_ is the offset. The *k*_d_app_early_ and *k*_d_app_late_ of the lectin nano-blocks were calculated using Equations (3) and (4), respectively.

The *k*_d_app_ of rACG was calculated using Equation (5) for the entire dissociation phase (181–780 s).

*R*_t_ = *R*_0_ exp(− *k*_d_app_ (*t* − *t*_0_)) (5)

## Figures and Tables

**Table 1:** Summary of the SEC–MALS results.

Sample (Peak)	Mass Fraction (%)	Molecular Mass (*M*_w_) (kDa)	*M*_w_/Theoretical *m* of a Monomer	Oligomeric State (mer)
WA20-HL4-ACG (i)	5.9	289	9.0	8, 10
WA20-HL4-ACG (ii)	16.3	214	6.7	6
WA20-HL4-ACG (iii)	30.4	125	3.9	4
WA20-HL4-ACG (iv)	47.3	61.6	1.9	2
WA20-FL4-ACG (i)	3.5	288	9.1	8, 10
WA20-FL4-ACG (ii)	7.0	190	6.0	6
WA20-FL4-ACG (iii)	22.7	121	3.8	4
WA20-FL4-ACG (iv)	66.8	59.8	1.9	2
WA20-SL-ACG (i)	5.3	339	11.3	10, 12
WA20-SL-ACG (ii)	6.5	261	8.7	8, 10
WA20-SL-ACG (iii)	11.8	185	6.1	6
WA20-SL-ACG (iv)	37.2	122	4.0	4
WA20-SL-ACG (v)	39.2	62.0	2.1	2
WA20-H-ACG (i)	19.1	310	10.4	10
WA20-H-ACG (ii)	11.6	237	8.0	8
WA20-H-ACG (iii)	16.7	173	5.8	6
WA20-H-ACG (iv)	36.1	118	4.0	4
WA20-H-ACG (v)	16.5	62.4	2.1	2
WA20-ΔN3ACG (i)	16.7	329	11.3	10, 12
WA20-ΔN3ACG (ii)	11.9	234	8.0	8
WA20-ΔN3ACG (iii)	19.0	169	5.8	6
WA20-ΔN3ACG (iv)	44.8	113	3.9	4
WA20-ΔN3ACG (v)	7.5	61.1	2.1	2

**Table 2:** Summary of the SAXS results.

Sample (Fraction)	*I*(*q*→0)/*c* (cm^−1^ mg^−1^ mL)	*D*_max_ (nm)	*R*_g_ (nm)	*M*_w_ (kDa)
WA20-SL-ACG (i)	0.2125	36.0	7.6	340
WA20-SL-ACG (ii)	0.1397	29.0	6.6	223
WA20-SL-ACG (iii)	0.0936	24.2	5.4	150
WA20-SL-ACG (iv)	0.0650	18.6	4.7	104
WA20-SL-ACG (v)	0.0378	14.9	3.4	60.5
WA20-H-ACG (i)	0.2003	36.0	8.3	320
WA20-H-ACG (ii)	0.1362	34.0	6.5	218
WA20-H-ACG (iii)	0.0971	30.6	6.3	155
WA20-H-ACG (iv)	0.0669	18.4	4.8	107
WA20-H-ACG (v)	0.0394	18.1	3.9	63.0
WA20-ΔN3ACG (i)	0.2046	34.0	8.1	327
WA20-ΔN3ACG (ii)	0.1370	29.0	6.5	219
WA20-ΔN3ACG (iii)	0.0996	23.2	5.5	159
WA20-ΔN3ACG (iv)	0.0689	17.4	4.5	110
WA20-ΔN3ACG (v)	0.0416	21.6	4.1	66.5
rACG	0.0199	6.8	2.2	31.8
WA20_H86K	0.0157	11.0	2.7	25.1
Ovalbumin *	0.0277	8.2	2.3	44.3

* Ovalbumin was used as a reference standard for molecular mass.

**Table 3:** MCA of the lectin nano-blocks.

Sample	MCA
WA20	No agglutination
rACG	42 nM
WA20-HL4-ACG	5.2 nM
WA20-FL4-ACG	1.3 nM
WA20-SL-ACG	1.3 nM
WA20-H-ACG	1.3 nM
WA20-ΔN3ACG	2.6 nM

**Table 4:** Summary of the SPR analysis results.

Sample (Fraction)	*R*_max_app_ (RU)	*K*_D_app_ (M)	*k*_d_app_early_ (s^−1^) (181–211 s)	*k*_d_app_late_ (s^−1^) (480–780 s)
WA20-H-ACG (I) (≥ decamer)	889.8	3.07 × 10^−7^	4.46 × 10^−2^	1.57 × 10^−4^
WA20-H-ACG (II) (octamer)	875.9	2.55 × 10^−7^	3.89 × 10^−2^	1.92 × 10^−4^
WA20-H-ACG (III) (hexamer)	799.4	2.84 × 10^−7^	5.23 × 10^−2^	2.83 × 10^−4^
WA20-H-ACG (IV) (tetramer)	675.5	7.40 × 10^−7^	6.69 × 10^−2^	5.49 × 10^−4^
WA20-H-ACG (V) (dimer)	556.8	1.36 × 10^−6^	1.02 × 10^−1^	3.62 × 10^−4^
WA20-ΔN3ACG (I) (≥ decamer)	937.7	3.75 × 10^−7^	4.49 × 10^−2^	1.46 × 10^−4^
WA20-ΔN3ACG (II) (octamer)	870.0	3.89 × 10^−7^	3.60 × 10^−2^	1.80 × 10^−4^
WA20-ΔN3ACG (III) (hexamer)	733.6	6.08 × 10^−7^	6.01 × 10^−2^	3.13 × 10^−4^
WA20-ΔN3ACG (IV) (tetramer)	613.5	1.12 × 10^−6^	7.80 × 10^−2^	7.27 × 10^−4^
WA20-ΔN3ACG (V)(dimer)	519.3	1.99 × 10^−6^	9.13 × 10^−2^	3.51 × 10^−4^
rACG (dimer)	490.6	1.97 × 10^−5^	1.14 × 10^−1^ (*k*_d_app_)	

## Data Availability

The structural models of the lectin nano-block oligomers in Figure 4 and Figure S19 and SAXS data are openly available in Small Angle Scattering Biological Data Bank (SASBDB) [56]: https://www.sasbdb.org (accessed on 7 January 2022) at accession codes: SASDNC3 for WA20-SL-ACG dimer, SASDND3 for WA20-SL-ACG tetramer, SASDNE3 for WA20-SL-ACG hexamer, SASDNF3 for WA20-H-ACG dimer, and SASDNG3 for WA20-ΔN3ACG dimer.

## Supplementary Materials

The following are available online at https://www.mdpi.com/article/10.3390/ijms23020676/s1.

## References

[1] Goodsell D.S., Olson A.J. (2000). Structural symmetry and protein function. Annu. Rev. Biophys. Biomol. Struct..

[2] Huang P.S., Boyken S.E., Baker D. (2016). The coming of age of de novo protein design. Nature.

[3] Arai R. (2018). Hierarchical design of artificial proteins and complexes toward synthetic structural biology. Biophys. Rev..

[4] Yeates T.O., Liu Y., Laniado J. (2016). The design of symmetric protein nanomaterials comes of age in theory and practice. Curr. Opin. Struct. Biol..

[5] Kobayashi N., Arai R. (2017). Design and construction of self-assembling supramolecular protein complexes using artificial and fusion proteins as nanoscale building blocks. Curr. Opin. Biotech..

[6] Hansen W.A., Khare S.D. (2020). Recent progress in designing protein-based supramolecular assemblies. Curr. Opin. Struct. Biol..

[7] Hecht M.H., Das A., Go A., Bradley L.H., Wei Y. (2004). De novo proteins from designed combinatorial libraries. Protein Sci..

[8] Patel S.C., Bradley L.H., Jinadasa S.P., Hecht M.H. (2009). Cofactor binding and enzymatic activity in an unevolved superfamily of de novo designed 4-helix bundle proteins. Protein Sci..

[9] Arai R., Kobayashi N., Kimura A., Sato T., Matsuo K., Wang A.F., Platt J.M., Bradley L.H., Hecht M.H. (2012). Domain-swapped dimeric structure of a stable and functional de novo four-helix bundle protein, WA20. J. Phys. Chem. B.

[10] Kimura N., Mochizuki K., Umezawa K., Hecht M.H., Arai R. (2020). Hyperstable de novo protein with a dimeric bisecting topology. ACS Synth. Biol..

[11] Irumagawa S., Kobayashi K., Saito Y., Miyata T., Umetsu M., Kameda T., Arai R. (2021). Rational thermostabilisation of four-helix bundle dimeric de novo proteins. Sci. Rep..

[12] Kobayashi N., Yanase K., Sato T., Unzai S., Hecht M.H., Arai R. (2015). Self-assembling nano-architectures created from a protein nano-building block using an intermolecularly folded dimeric de novo protein. J. Am. Chem. Soc..

[13] Kobayashi N., Inano K., Sasahara K., Sato T., Miyazawa K., Fukuma T., Hecht M.H., Song C., Murata K., Arai R. (2018). Self-assembling supramolecular nanostructures constructed from de novo extender protein nanobuilding blocks. ACS Synth. Biol..

[14] Bonnardel F., Mariethoz J., Salentin S., Robin X., Schroeder M., Perez S., Lisacek F., Imberty A. (2019). UniLectin3D, a database of carbohydrate binding proteins with curated information on 3D structures and interacting ligands. Nucleic Acids Res..

[15] Ward E.M., Kizer M.E., Imperiali B. (2021). Strategies and tactics for the development of selective glycan-binding proteins. ACS Chem. Biol..

[16] Varki A. (2017). Biological roles of glycans. Glycobiology.

[17] Arnaud J., Audfray A., Imberty A. (2013). Binding sugars: From natural lectins to synthetic receptors and engineered neolectins. Chem. Soc. Rev..

[18] Hirabayashi J., Arai R. (2019). Lectin engineering: The possible and the actual. Interface Focus.

[19] Ribeiro J.P., Villringer S., Goyard D., Coche-Guerente L., Hoferlin M., Renaudet O., Romer W., Imberty A. (2018). Tailor-made Janus lectin with dual avidity assembles glycoconjugate multilayers and crosslinks protocells. Chem. Sci..

[20] Kitaguchi D., Oda T., Enomoto T., Ohara Y., Owada Y., Akashi Y., Furuta T., Yu Y., Kimura S., Kuroda Y. (2020). Lectin drug conjugate therapy for colorectal cancer. Cancer Sci..

[21] Coves-Datson E.M., King S.R., Legendre M., Gupta A., Chan S.M., Gitlin E., Kulkarni V.V., Garcia J.P., Smee D.F., Lipka E. (2020). A molecularly engineered antiviral banana lectin inhibits fusion and is efficacious against influenza virus infection in vivo. Proc. Natl. Acad. Sci. USA.

[22] Meany D.L., Zhang Z., Sokoll L.J., Zhang H., Chan D.W. (2009). Glycoproteomics for prostate cancer detection: Changes in serum PSA glycosylation patterns. J. Proteome Res..

[23] Weis W.I., Drickamer K. (1996). Structural basis of lectin-carbohydrate recognition. Annu. Rev. Biochem..

[24] Notova S., Bonnardel F., Lisacek F., Varrot A., Imberty A. (2020). Structure and engineering of tandem repeat lectins. Curr. Opin. Struct. Biol..

[25] Yabe R., Itakura Y., Nakamura-Tsuruta S., Iwaki J., Kuno A., Hirabayashi J. (2009). Engineering a versatile tandem repeat-type alpha2-6sialic acid-binding lectin. Biochem. Biophys. Res. Commun..

[26] Mahajan S., Ramya T.N.C. (2018). Nature-inspired engineering of an F-type lectin for increased binding strength. Glycobiology.

[27] Hamorsky K.T., Kouokam J.C., Dent M.W., Grooms T.N., Husk A.S., Hume S.D., Rogers K.A., Villinger F., Morris M.K., Hanson C.V. (2019). Engineering of a lectibody targeting high-mannose-type glycans of the HIV envelope. Mol. Ther..

[28] Yagi F., Miyamoto M., Abe T., Minami Y., Tadera K., Goldstein I.J. (1997). Purification and carbohydrate-binding specificity of *Agrocybe cylindracea* lectin. Glycoconj. J..

[29] Yagi F., Hiroyama H., Kodama S. (2001). *Agrocybe cylindracea* lectin is a member of the galectin family. Glycoconj. J..

[30] Ban M., Yoon H.J., Demirkan E., Utsumi S., Mikami B., Yagi F. (2005). Structural basis of a fungal galectin from *Agrocybe cylindracea* for recognizing sialoconjugate. J. Mol. Biol..

[31] Imamura K., Takeuchi H., Yabe R., Tateno H., Hirabayashi J. (2011). Engineering of the glycan-binding specificity of *Agrocybe cylindracea* galectin towards α(2,3)-linked sialic acid by saturation mutagenesis. J. Biochem..

[32] Hu D., Tateno H., Hirabayashi J. (2015). Lectin engineering, a molecular evolutionary approach to expanding the lectin utilities. Molecules.

[33] Hu D., Tateno H., Sato T., Narimatsu H., Hirabayashi J. (2013). Tailoring GalNAcα1-3Galβ-specific lectins from a multi-specific fungal galectin: Dramatic change of carbohydrate specificity by a single amino-acid substitution. Biochem. J..

[34] Hu D., Huang H., Tateno H., Nakakita S.-i., Sato T., Narimatsu H., Yao X., Hirabayashi J. (2015). Engineering of a 3′-sulpho-Galβ1-4GlcNAc-specific probe by a single amino acid substitution of a fungal galectin. J. Biochem..

[35] Hirabayashi J., Hu D., Tateno H., Kuwabara N., Kato R., Yagi F. (2018). Carbohydrate recognition mechanism of the mushroom galectin ACG. Trends Glycosci. Glycotech..

[36] Arai R. (2021). Design of helical linkers for fusion proteins and protein-based nanostructures. Methods Enzymol..

[37] Arai R., Ueda H., Kitayama A., Kamiya N., Nagamune T. (2001). Design of the linkers which effectively separate domains of a bifunctional fusion protein. Protein Eng..

[38] Arai R., Wriggers W., Nishikawa Y., Nagamune T., Fujisawa T. (2004). Conformations of variably linked chimeric proteins evaluated by synchrotron X-ray small-angle scattering. Proteins.

[39] Petoukhov M.V., Franke D., Shkumatov A.V., Tria G., Kikhney A.G., Gajda M., Gorba C., Mertens H.D., Konarev P.V., Svergun D.I. (2012). New developments in the ATSAS program package for small-angle scattering data analysis. J. Appl. Crystallogr..

[40] Tateno H., Mori A., Uchiyama N., Yabe R., Iwaki J., Shikanai T., Angata T., Narimatsu H., Hirabayashi J. (2008). Glycoconjugate microarray based on an evanescent-field fluorescence-assisted detection principle for investigation of glycan-binding proteins. Glycobiology.

[41] Munkley J. (2019). The glycosylation landscape of pancreatic cancer. Oncol. Lett..

[42] Munoz E.M., Correa J., Riguera R., Fernandez-Megia E. (2013). Real-time evaluation of binding mechanisms in multivalent interactions: A surface plasmon resonance kinetic approach. J. Am. Chem. Soc..

[43] Erijman A., Dantes A., Bernheim R., Shifman J.M., Peleg Y. (2011). Transfer-PCR (TPCR): A highway for DNA cloning and protein engineering. J. Struct. Biol..

[44] Pace C.N., Vajdos F., Fee L., Grimsley G., Gray T. (1995). How to measure and predict the molar absorption coefficient of a protein. Protein Sci..

[45] Matsumoto I., Mizuno Y., Seno N. (1979). Activation of Sepharose with epichlorohydrin and subsequent immobilization of ligand for affinity adsorbent. J. Biochem..

[46] Wyatt P.J. (1993). Light-scattering and the absolute characterization of macromolecules. Anal. Chim. Acta.

[47] Shimizu N., Mori T., Nagatani Y., Ohta H., Saijo S., Takagi H., Takahashi M., Yatabe K., Kosuge T., Igarashi N. (2019). BL-10C, the small-angle x-ray scattering beamline at the photon factory. AIP Conf. Proc..

[48] Shimizu N., Yatabe K., Nagatani Y., Saijyo S., Kosuge T., Igarashi N. (2016). Software development for analysis of small-angle X-ray scattering data. AIP Conf. Proc..

[49] Svergun D.I. (1992). Determination of the regularization parameter in indirect-transform methods using perceptual criteria. J. Appl. Crystallogr..

[50] Franke D., Petoukhov M.V., Konarev P.V., Panjkovich A., Tuukkanen A., Mertens H.D.T., Kikhney A.G., Hajizadeh N.R., Franklin J.M., Jeffries C.M. (2017). ATSAS 2.8: A comprehensive data analysis suite for small-angle scattering from macromolecular solutions. J. Appl. Crystallogr..

[51] Glatter O., Kratky O. (1982). Small-Angle X-Ray Scattering.

[52] Franke D., Svergun D.I. (2009). DAMMIF, a program for rapid ab-initio shape determination in small-angle scattering. J. Appl. Crystallogr..

[53] Volkov V.V., Svergun D.I. (2003). Uniqueness of ab initio shape determination in small-angle scattering. J. Appl. Crystallogr..

[54] Svergun D.I. (1999). Restoring low resolution structure of biological macromolecules from solution scattering using simulated annealing. Biophys. J..

[55] Pettersen E.F., Goddard T.D., Huang C.C., Couch G.S., Greenblatt D.M., Meng E.C., Ferrin T.E. (2004). UCSF Chimera—A visualization system for exploratory research and analysis. J. Comput. Chem..

[56] Kikhney A.G., Borges C.R., Molodenskiy D.S., Jeffries C.M., Svergun D.I. (2020). SASBDB: Towards an automatically curated and validated repository for biological scattering data. Protein Sci..

