# Supplementary material for: Self-Assembling Lectin Nano-Block Oligomers Enhance Binding Avidity to Glycans
Source: Int J Mol Sci. 2022 Jan 8;23(2):676. doi: 10.3390/ijms23020676 (PMC8775495; doi:10.3390/ijms23020676)
Supplement: Supplementary file 1 [file ijms-23-00676-s001.zip › ijms-1527006-supplementary.pdf]

Supplementary Materials

Self-Assembling Lectin Nano-Block Oligomers Enhance Binding Avidity to Glycans

Shin Irumagawa[1,2,3], Keiko Hiemori [4], Sayoko Saito [4], Hiroaki Tateno[4] and Ryoichi Arai[1,2,3,*]

[1] Department of Biomolecular Innovation, Institute for Biomedical Sciences, Interdisciplinary Cluster for Cutting Edge Research, Shinshu University, Ueda, Nagano 386-8567, Japan
[2] Department of Applied Biology, Faculty of Textile Science and Technology, Shinshu University, Ueda, Nagano 386-8567, Japan
[3] Department of Science and Technology, Graduate School of Medicine, Science and Technology, Shinshu University, Ueda, Nagano 386-8567, Japan
[4] Cellular and Molecular Biotechnology Research Institute, National Institute of Advanced Industrial Science and Technology (AIST), Tsukuba, Ibaraki 305-8566, Japan

* Correspondence: rarai@shinshu-u.ac.jp

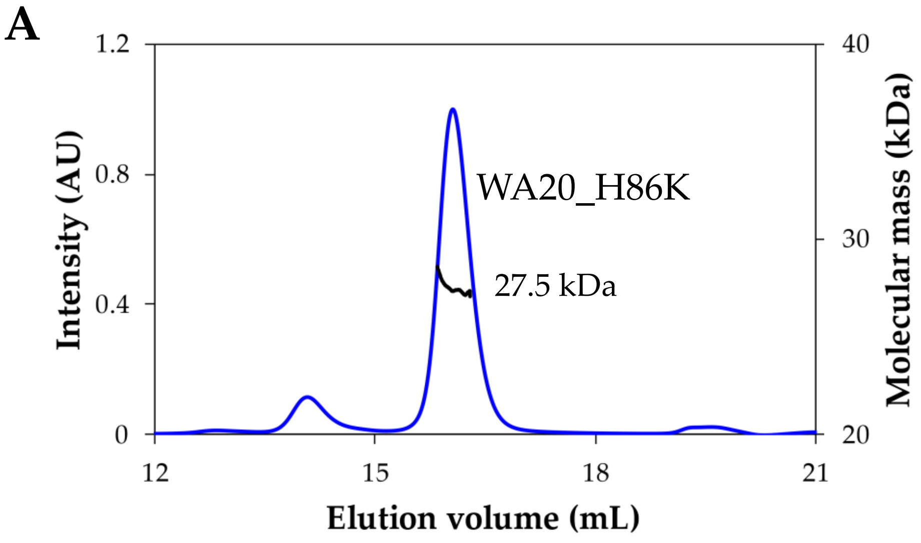

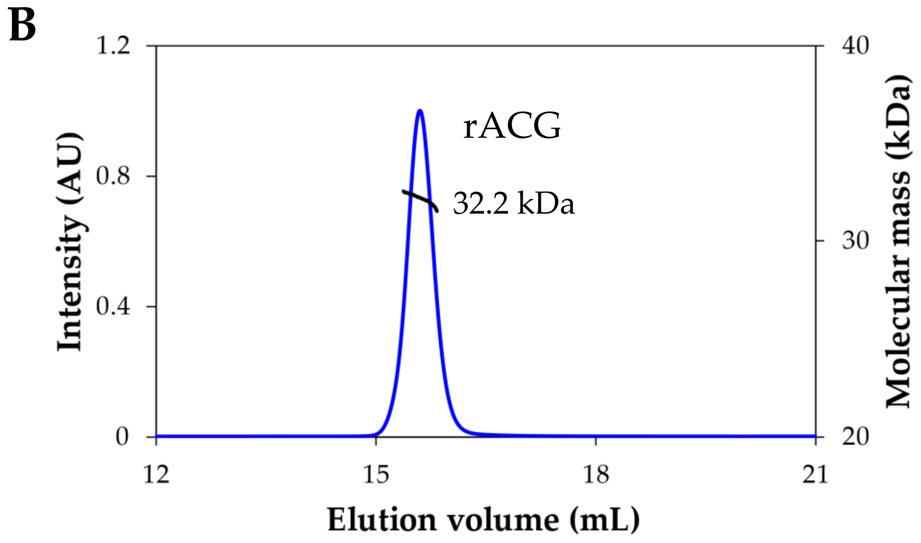

Figure S1. Size exclusion chromatography–multi angle light scattering (SEC–MALS) profiles of (A) de novo protein WA20_H86K mutant and (B) recombinant *Agrocybe cylindracea* galectin (rACG).

The blue and black lines represent the normalized intensity of UV absorbance (A_{280nm}) and the molecular mass of the protein, respectively. The molecular mass values of WA20_H86K and rACG were calculated to be 27.5 kDa and 32.2 kDa, respectively. Because the theoretical molecular mass values of a WA20_H86K monomer and a rACG monomer are 12.5 kDa and 17.0 kDa, respectively, Both proteins formed dimers in solution.

WA20-HL4-ACG (*m*: 32.1 kDa)

```
MYGKLNKLVE HIKELLQQLN KNWHRHQGNL HDMNQQMEQL FQEFQHFMQG
NQDDGKLQNM IHEMQQFMNQ VDNHLQSESD TVHHFKNKLQ ELMNNFHHLV
HRKLAEAAAK EAAAKEAAAK EAAAKAAAHM TTSAVNIYNI SAGASVDLAA
PVTTGDIVTF FSSALNLSAG AGSPNNTALN LLSENGAYLL HIAFRLQENV
IVFNSRQPNA PWLVEQRVSN VANQFIGSGG KAMVTVFDHG DKYQVVINEK
TVIQYTKQIS GTTSSLSYNS TEGTSIFSTV VEAVTYTGLA
```

WA20-FL4-ACG (*m*: 31.5 kDa)

```
MYGKLNKLVE HIKELLQQLN KNWHRHQGNL HDMNQQMEQL FQEFQHFMQG
NQDDGKLQNM IHEMQQFMNQ VDNHLQSESD TVHHFKNKLQ ELMNNFHHLV
HRKLSGGGGS GGGGSGGGGS GGGGSAAAHM TTSAVNIYNI SAGASVDLAA
PVTTGDIVTF FSSALNLSAG AGSPNNTALN LLSENGAYLL HIAFRLQENV
IVFNSRQPNA PWLVEQRVSN VANQFIGSGG KAMVTVFDHGDKYQVVINEK
TVIQYTKQIS GTTSSLSYNS TEGTSIFSTV VEAVTYTGLA
```

WA20-SL-ACG (*m*: 30.1 kDa)

```
MYGKLNKLVE HIKELLQQLN KNWHRHQGNL HDMNQQMEQL FQEFQHFMQG
NQDDGKLQNM IHEMQQFMNQ VDNHLQSESD TVHHFKNKLQ ELMNNFHHLV
HRKLAAAHMT TSAVNIYNIS AGASVDLAAP VTTGDIVTFF SSALNLSAGA
GSPNNTALNL LSENGAYLLH IAFRLQENVI VFNSRQPNAP WLVEQRVSNV
ANQFIGSGGK AMVTVFDHGD KYQVVINEKT VIQYTKQISG TTSSLSYNST
EGTSIFSTVV EAVTYTGLA
```

WA20-H-ACG (*m*: 29.7 kDa)

```
MYGKLNKLVE HIKELLQQLN KNWHRHQGNL HDMNQQMEQL FQEFQHFMQG
NQDDGKLQNM IHEMQQFMNQ VDNHLQSESD TVHHFKNKLQ ELMNNFHHLV
HRHMTTSAVN IWNISAGASV DLAAPVTTGD IVTFFSSALN LSAGAGSPNN
TALNLLSENG AYLLHIAFRL QENVIVFNSR QPNAPWLVEQ RVSNVANQFI
GSGGKAMVTV FDHGDKYQVV INEKTVIQYT KQISGTTSSL SYNSTEGTSI
FSTVVEAVTY TGLA
```

WA20-ΔN3ACG (*m*: 29.2 kDa)

```
MYGKLNKLVE HIKELLQQLN KNWHRHQGNL HDMNQQMEQL FQEFQHFMQG
NQDDGKLQNM IHEMQQFMNQ VDNHLQSESD TVHHFKNKLQ ELMNNFHHLV
HRSAVNIYNI SAGASVDLAA PVTTGDIVTF FSSALNLSAG AGSPNNTALN
LLSENGAYLL HIAFRLQENV IVFNSRQPNA PWLVEQRVSN VANQFIGSGG
KAMVTVFDHG DKYQVVINEK TVIQYTKQIS GTTSSLSYNS TEGTSIFSTV
VEAVTYTGLA
```

Figure S2. Amino acid sequences of lectin nano-blocks.
Blue, red, and black letters represent the sequences of WA20_H86K, ACG, and peptide linker, respectively. The *m* means the theoretical molecular mass of a monomer of each lectin nano-block.

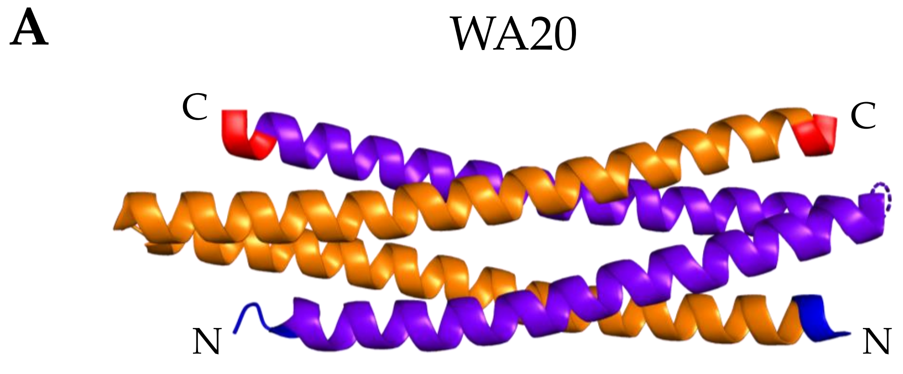

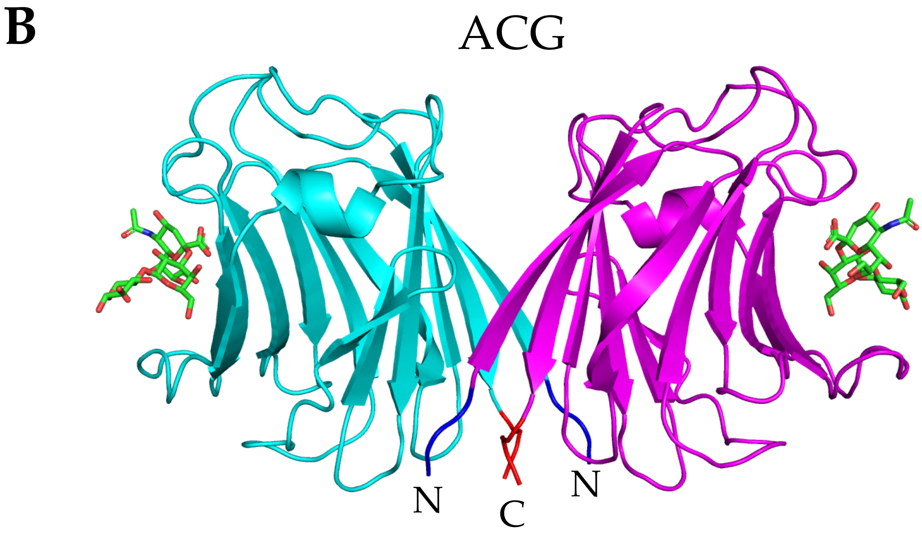

Figure S3. Crystal structures of WA20 and ACG.
The dimer structures of (**A**) WA20 (PDB ID: 3VJF) [9] and (B) ACG (PDB ID: 1WW4) [30]. (A) Chains A and B of WA20 are shown in orange and purple, respectively. The residues of N-termini (chain A: 3−5, chain B: 5−7) and C-termini (chain A and B: 99−101) are shown in blue and red, respectively. (**B**) Chains A and D of ACG are shown in magenta and cyan, respectively. Two NeuAc*α*2-3lactose shown as sticks. The residues of N-termini (chain A and D: 2−4) and C-termini (chain A and D: 159−161) residues are shown in blue and red, respectively.

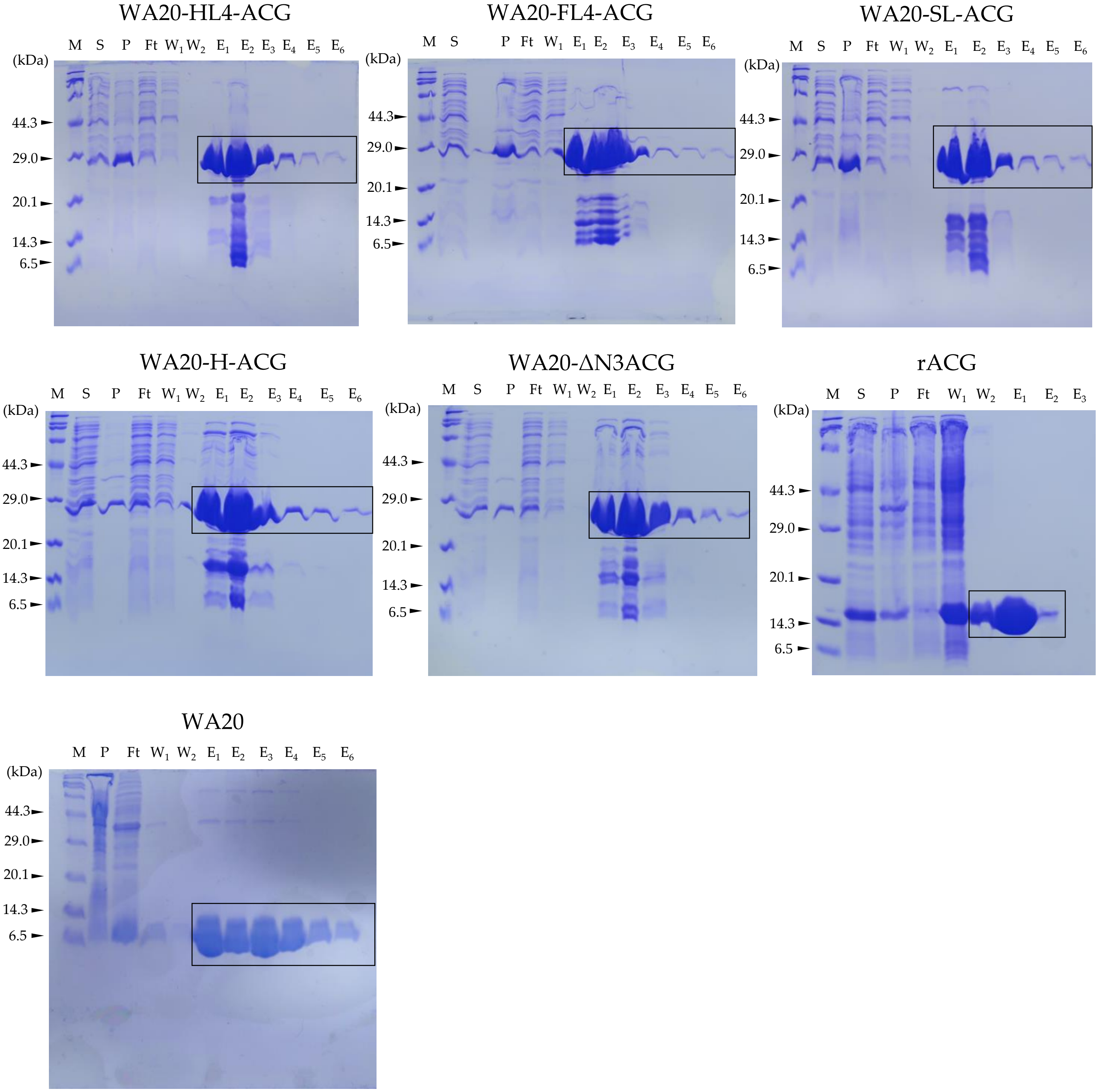

Figure S4. Sodium dodecyl sulfate polyacrylamide gel electrophoresis (SDS-PAGE) analysis. SDS-PAGE analysis of the five lectin nano-blocks and WA20 were performed for the eluted fractions by immobilized metal ion affinity chromatography (IMAC) with TALON metal affinity resin (Takara Bio, Kusatsu, Shiga, Japan), and recombinant ACG (rACG) was performed for the eluted fractions by lactose-immobilized Sepharose column. The protein bands in black rectangles show purified proteins. Protein molecular weight markers (broad) (Takara Bio) are shown in the far-left lane. Proteins were stained with Coomassie brilliant blue. Abbreviations: M, molecular weight marker; S, supernatant fraction; P, pellet fraction; Ft, flow-through fraction; W, wash fractions; E, eluted fractions.

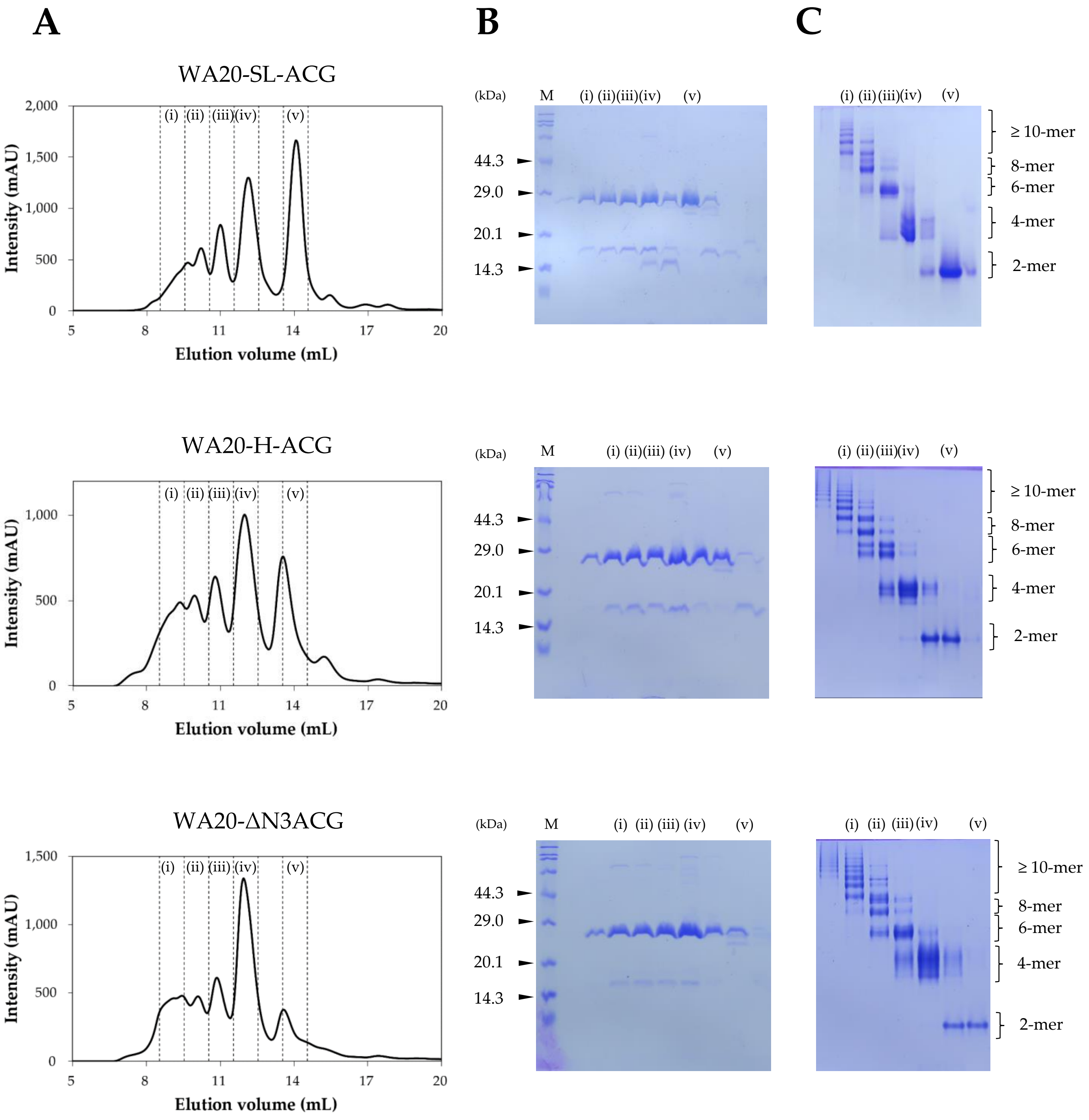

Figure S5. Size exclusion chromatography (SEC) purification of lectin nano-blocks for small-angle X-ray scattering (SAXS) experiments.
(**A**) SEC purification profiles of the IMAC-purified samples of WA20-SL-ACG (upper panel), WA20-H-ACG (middle panel), and WA20-ΔN3ACG (lower panel) with a Superdex 200 increase 10/300 GL column (Cytiva). The eluted fractions, Fr. (i), (ii), (iii), (iv), and (v) were used as samples for SAXS experiments of the lectin nano-block oligomers.
(**B**) SDS-PAGE and (**C**) native PAGE were performed for the fractionated samples. Proteins were stained with Coomassie brilliant blue. The oligomeric states of the protein bands in native PAGE were estimated from the SEC–MALS results.

A

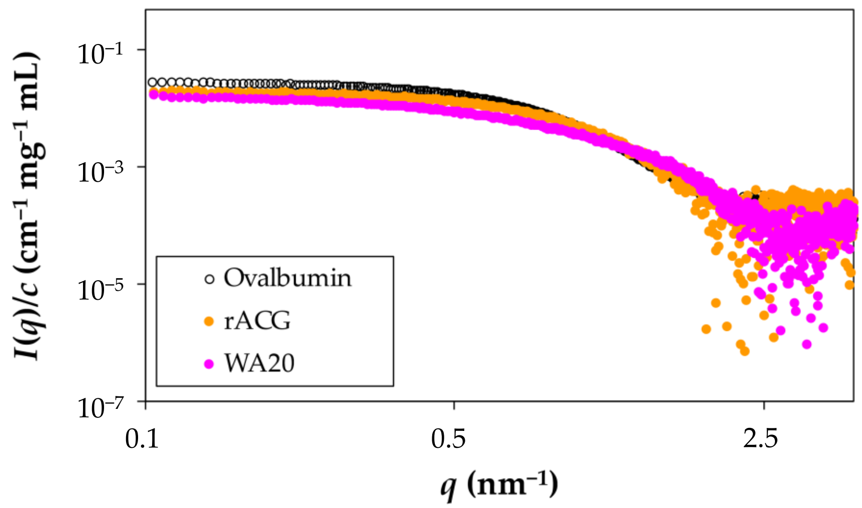

B

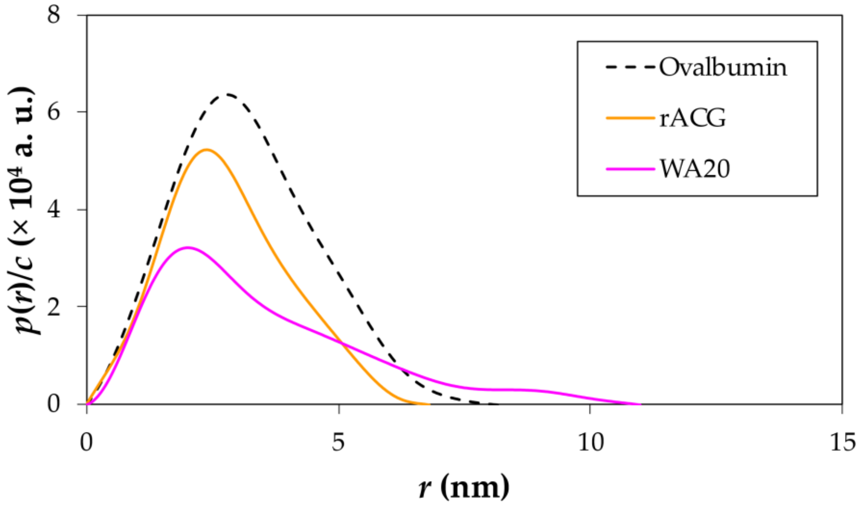

Figure S6. SAXS analysis of WA20, rACG, and ovalbumin.
(**A**) Concentration-normalized absolute scattering intensities of ovalbumin, WA20, and rACG. Ovalbumin was used as a reference standard of the molecular mass.
(**B**) Pair-distance distribution functions normalized by the concentration, $p(r)/c$, are obtained by inverse Fourier transformation.

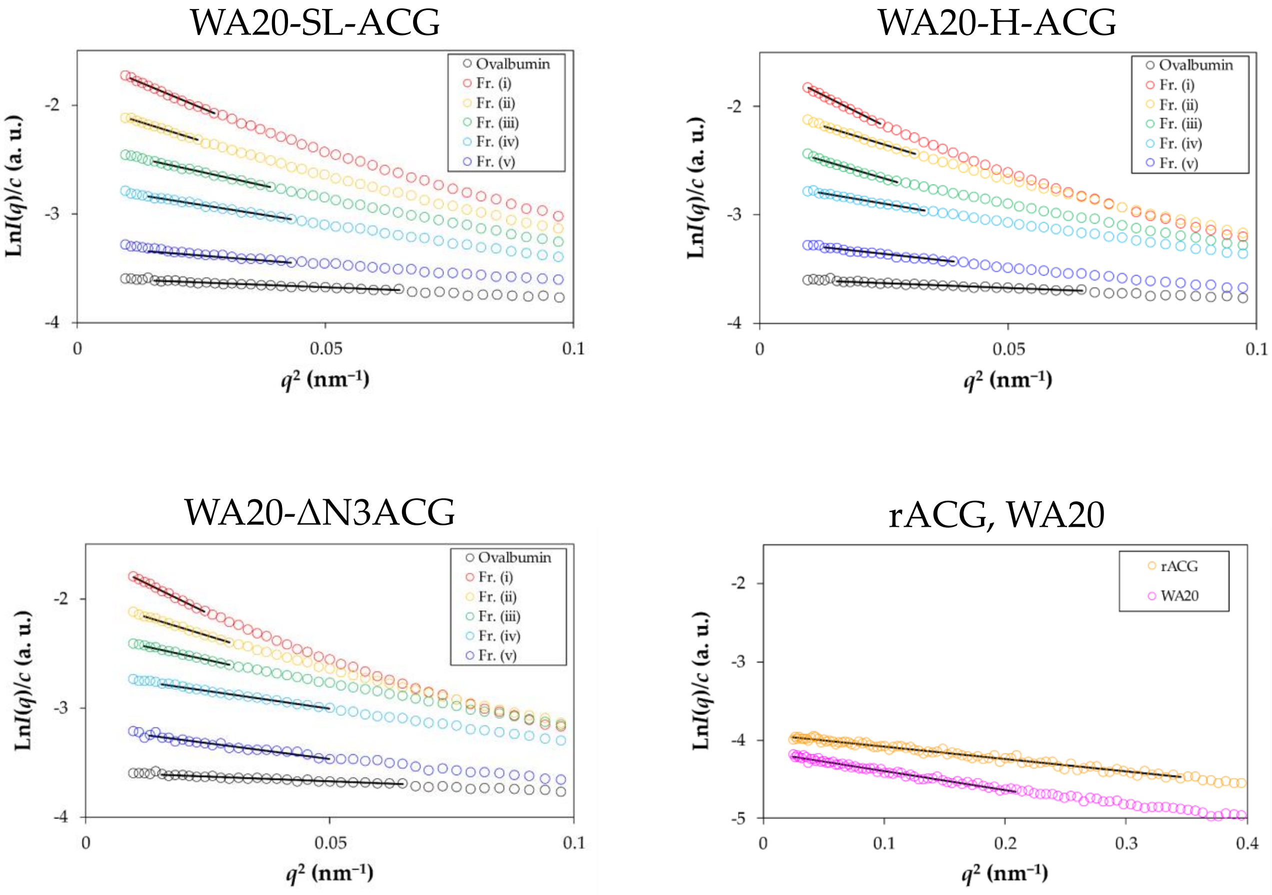

Figure S7. Guinier plots of the lectin nano-blocks, rACG, and WA20.
Forward scattering intensity $I(q \rightarrow 0)$ and radius of gyration R_g of the lectin nano-block oligomers were estimated based on their SAXS data by Guinier approximation using the AUTORG program in ATSAS [50] with SAngler [48].

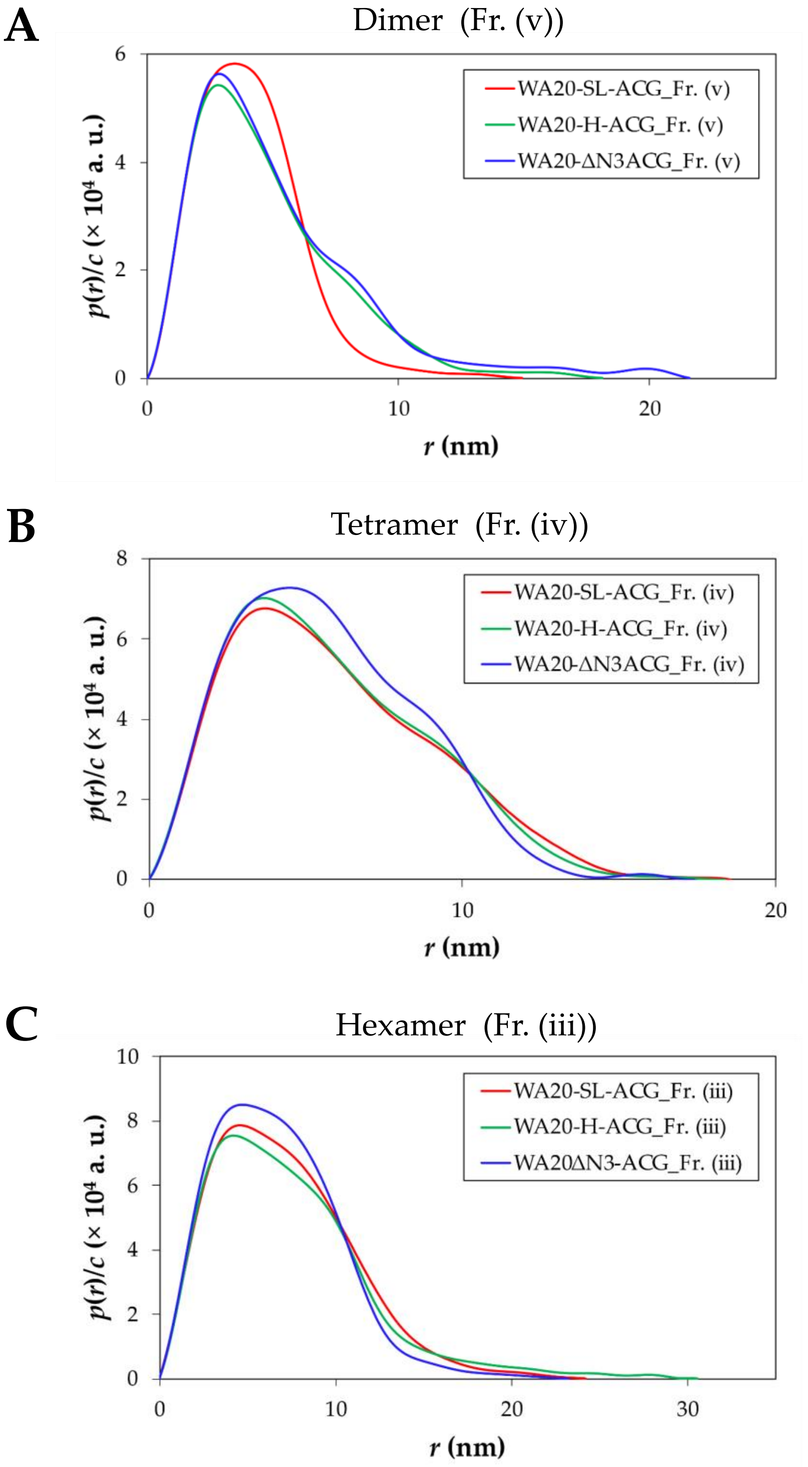

Figure S8. Comparisons of the $p(r)$ functions for (**A**) dimer, (**B**) tetramer, and (**C**) hexamer of the lectin nano-blocks.

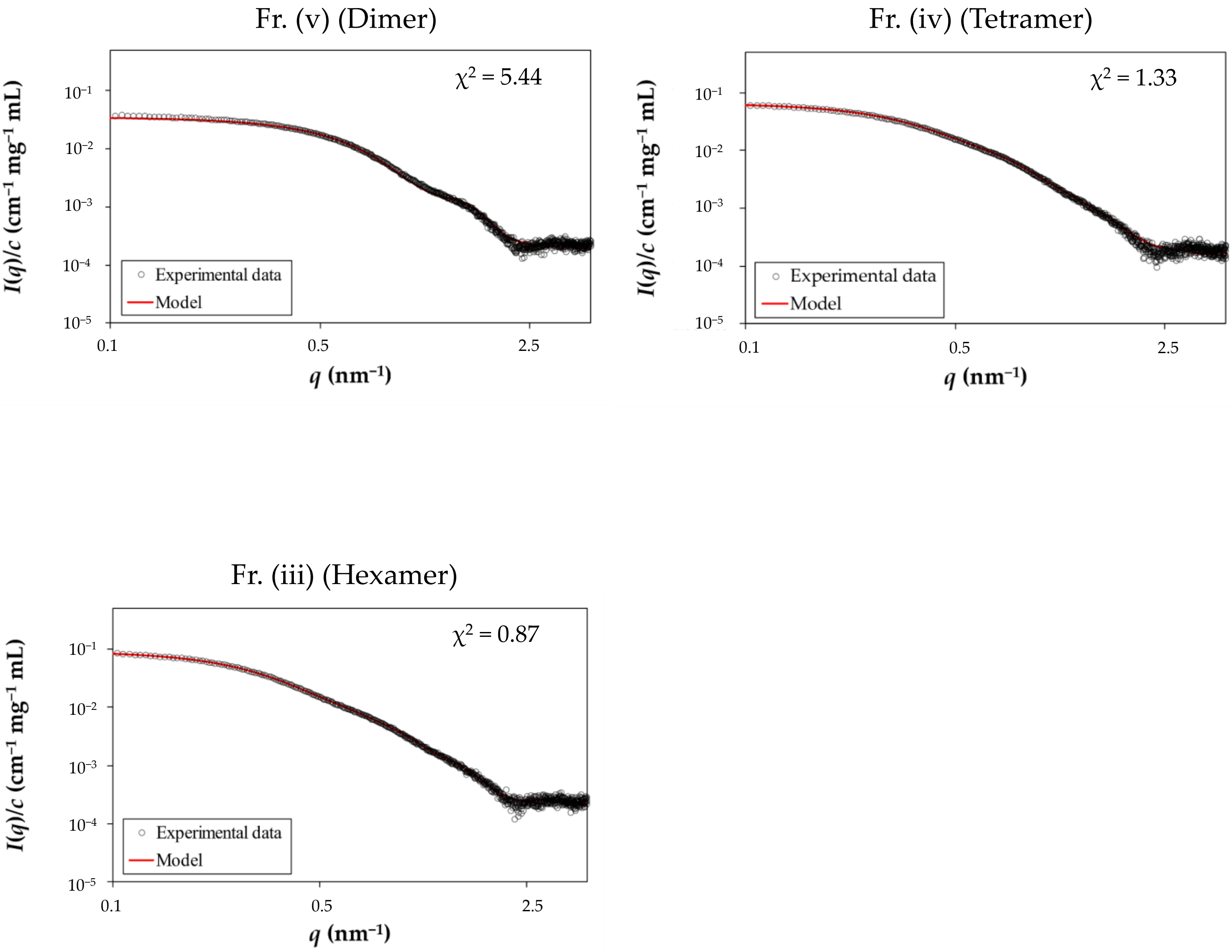

Figure S9. Plots of the scattering curves calculated from the CORAL models of WA20-SL-ACG oligomers fitting to the experimental SAXS data.
The concentration-normalized SAXS intensity $I(q)/c$ of the fractionated samples (Fr.) of WA20-SL-ACG (black open circle) and that optimized by the CORAL procedure (red line). The χ^2 value represents the degree of fitting between the experimental data and the data calculated from the CORAL model.

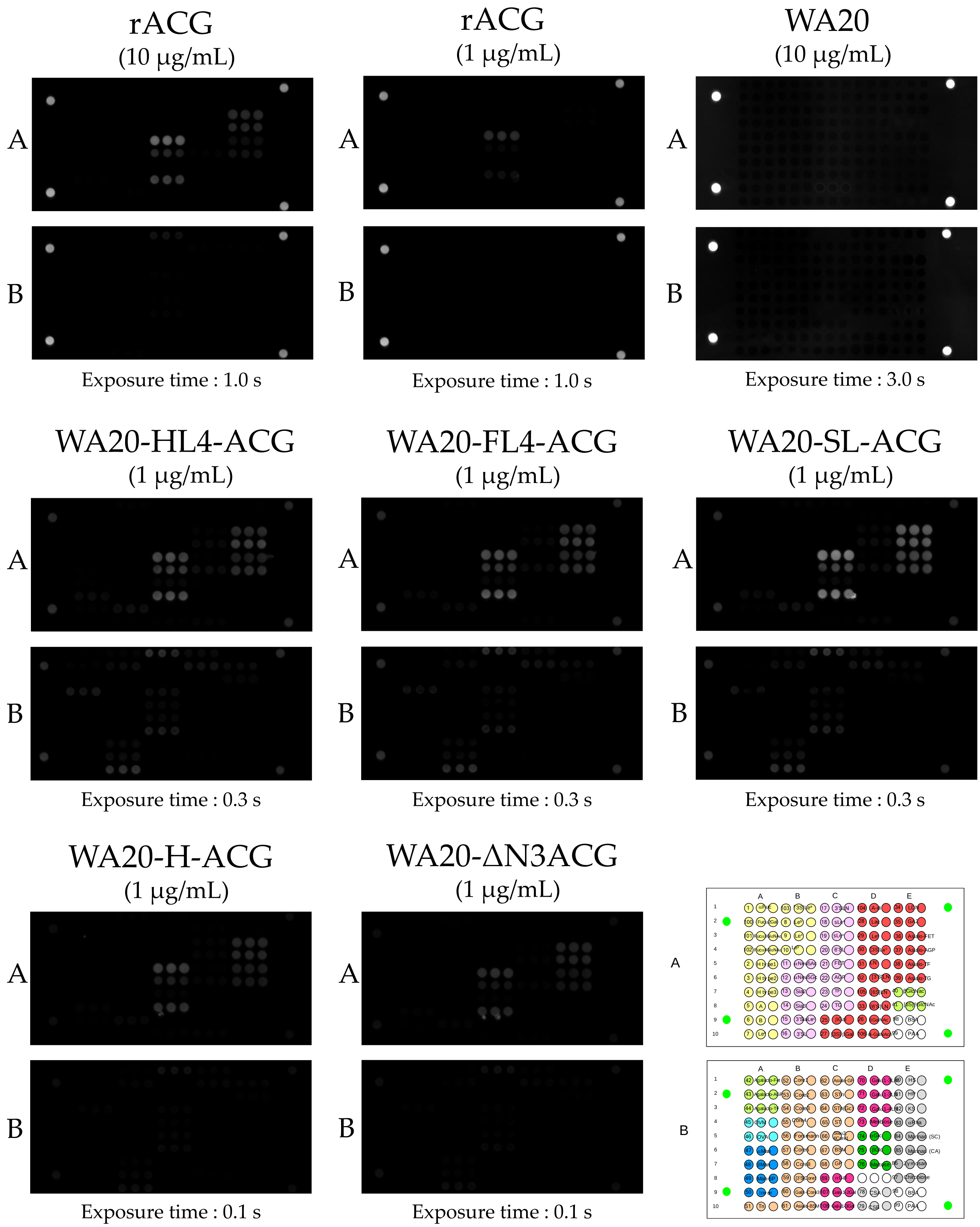

Figure S10. Fluorescence images of glycoconjugate microarray experiments.
The list of glycans used for glycoconjugate microarray analysis is shown in Table S1.

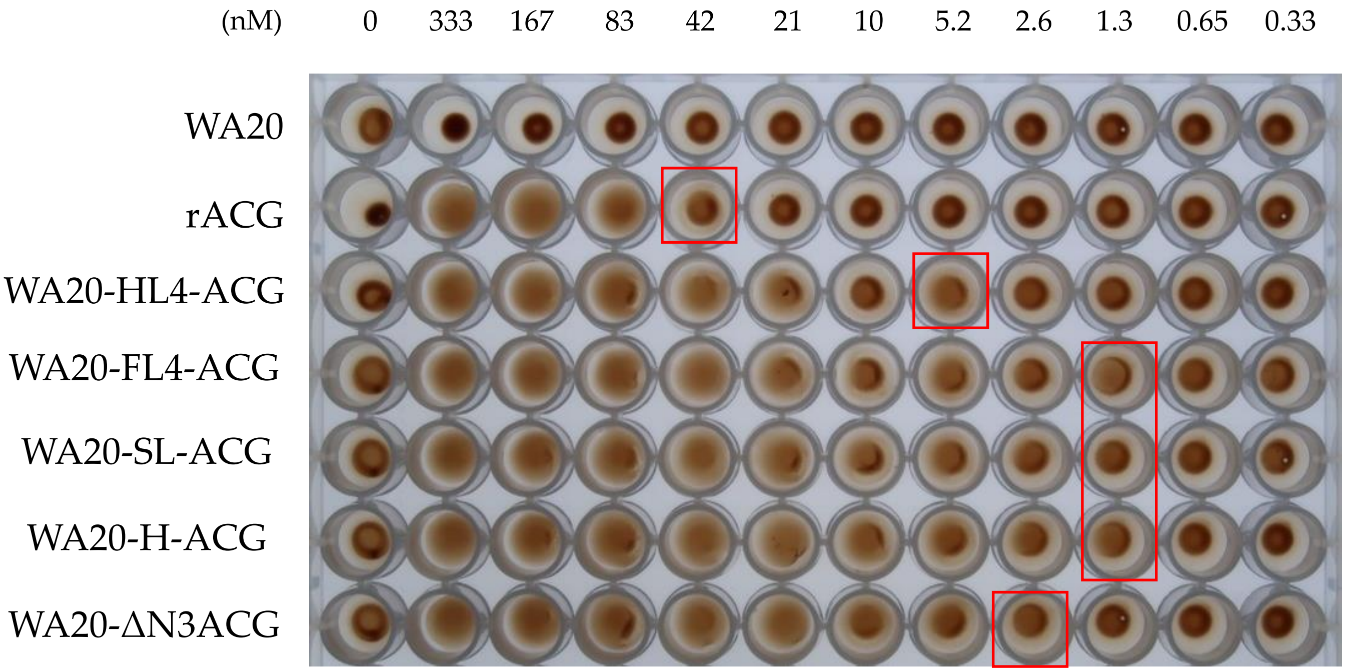

Figure S11. Hemagglutination assay results.
Hemagglutination assay was performed for WA20, rACG, and the five lectin nano-blocks. The wells surrounded by red frames show the minimum concentration of the samples with hemagglutination activity.

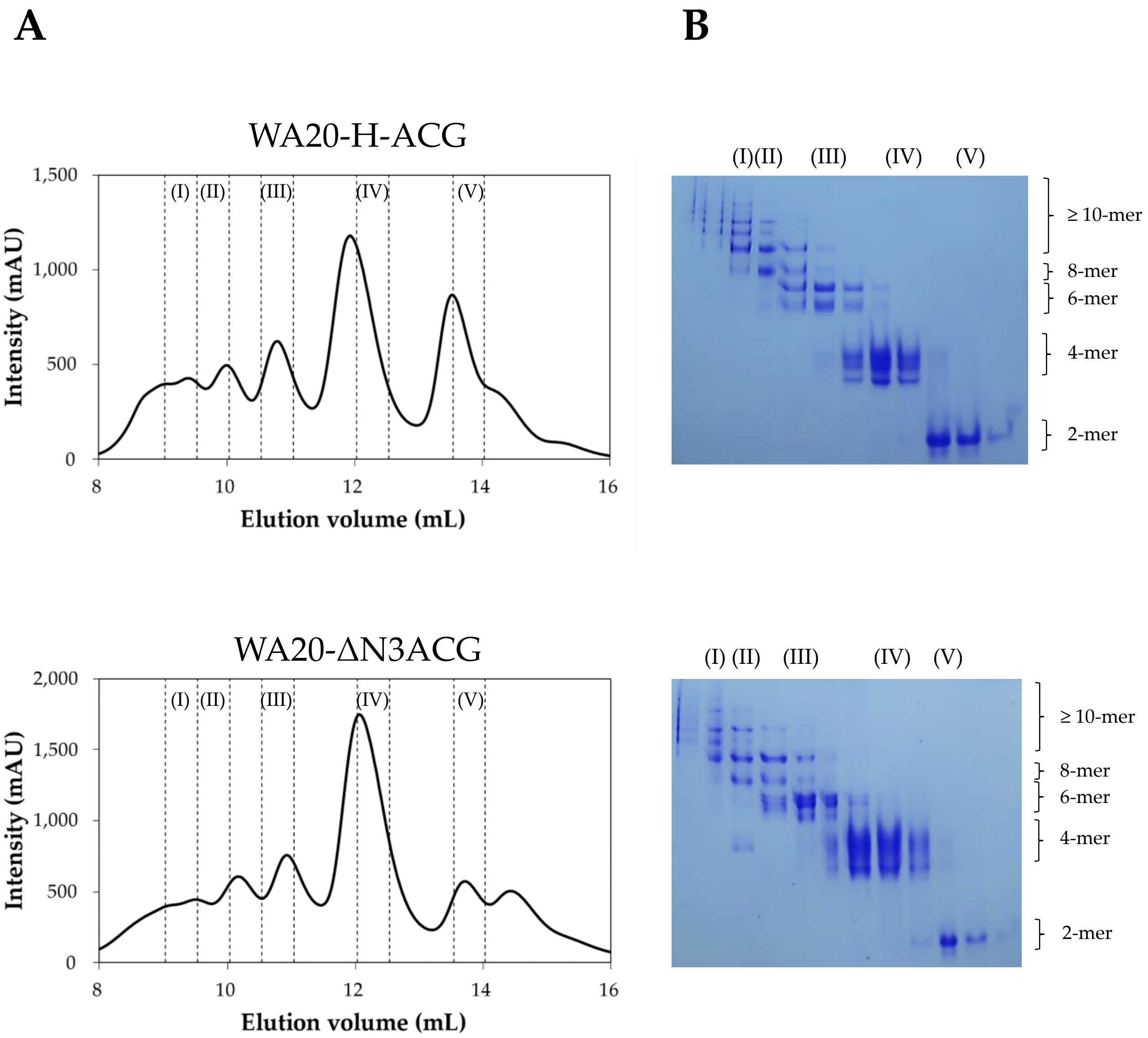

Figure S12. SEC purification of lectin nano-blocks for surface plasmon resonance (SPR) experiments.
(**A**) SEC purification of the IMAC-purified samples of the lectin nano-blocks WA20-H-ACG (upper panel) and WA20-ΔN3ACG (lower panel) with a Superdex 200 increase 10/300 GL column (Cytiva). The eluted fractions (I), (II), (III), (IV), and (V) were used as the samples of the lectin nano-block oligomers for SPR experiments. (**B**) Native PAGE analysis of the eluted fractions.

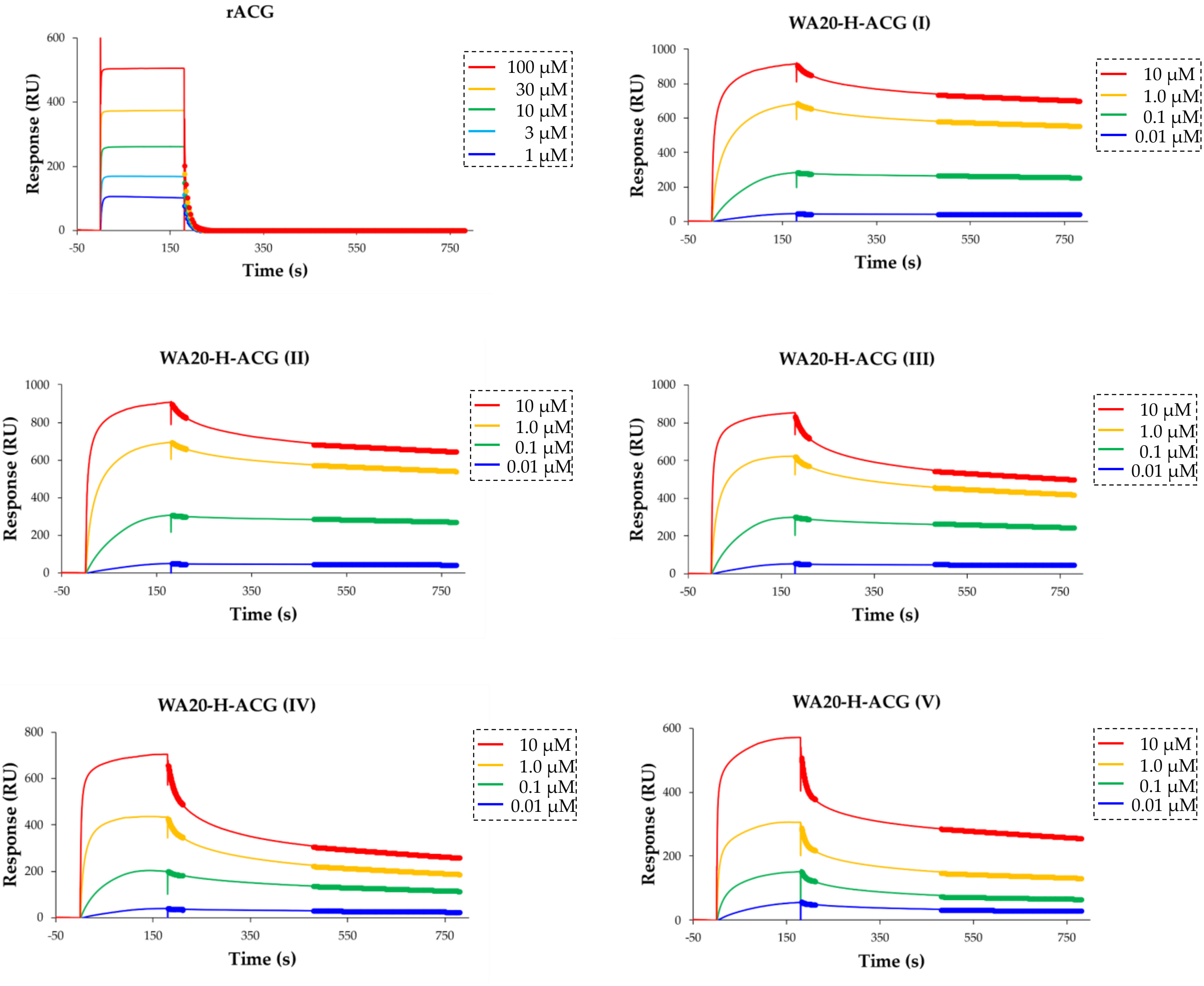

Figure S13. SPR analysis of the WA20-H-ACG oligomers and rACG. Sensorgrams of the WA20-H-ACG oligomers and rACG at several concentrations, bound to immobilized Neu5Acα2-3Galβ1-4Glcβ-Gly-PAA-biotin ligands. Bold lines show the fitting curves used for calculating the apparent dissociation rate constants $k_{\mathrm{d\_app\_early}}$ in the early phase (181–211 s) and $k_{\mathrm{d\_app\_late}}$ in the late phase (480–780 s) of the lectin nano-block oligomers. The apparent dissociation rate constant $k_{\mathrm{d\_app}}$ of rACG was calculated using the data in the entire dissociation time (181–780 s).

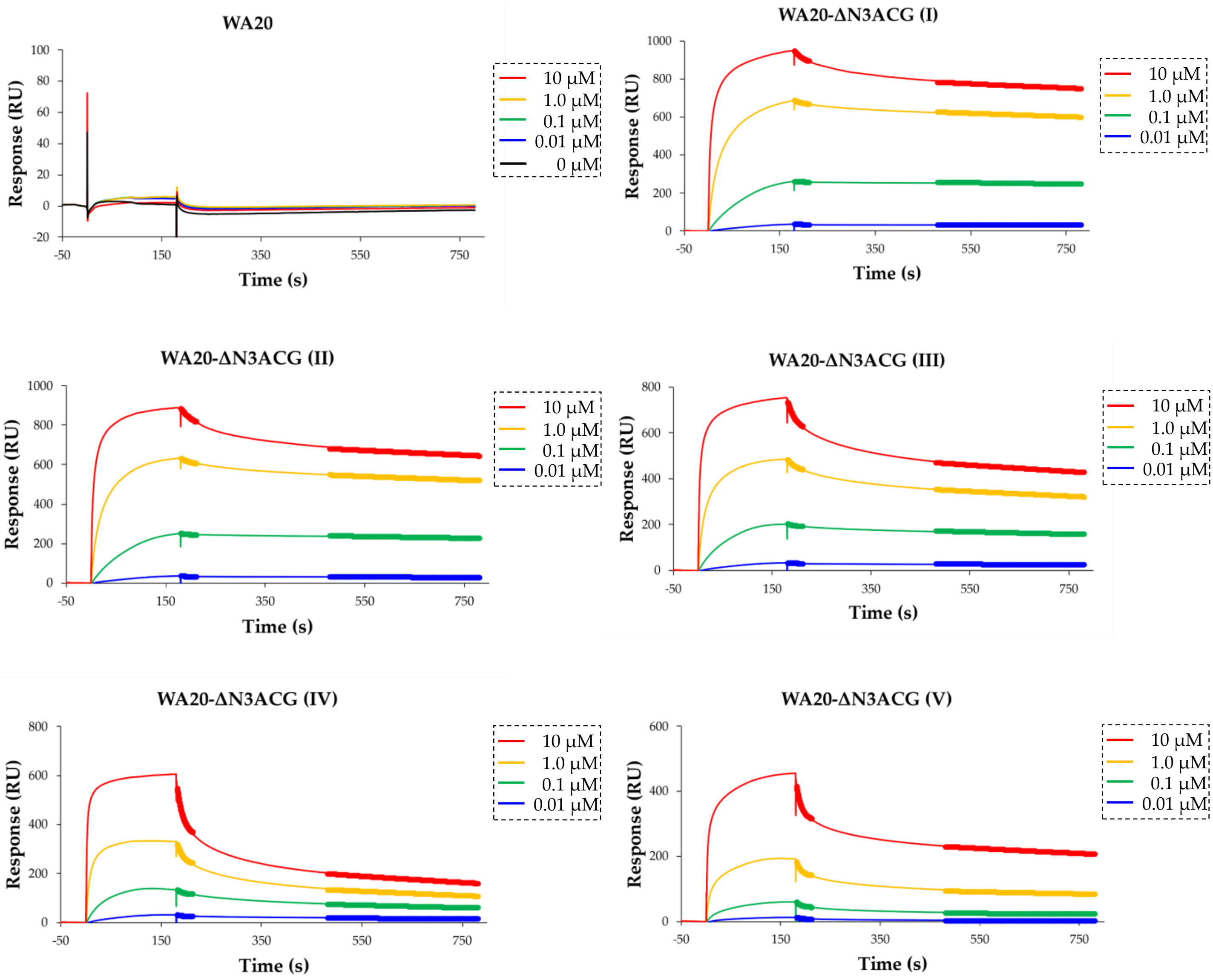

Figure S14. SPR analysis of the WA20-ΔN3ACG oligomers and WA20. Sensorgrams of the WA20-ΔN3ACG oligomers and WA20 at several concentrations, bound to immobilized Neu5Acα2-3Galβ1-4Glcβ-Gly-PAA-biotin ligands. Bold lines show the fitting curves used for calculating the apparent dissociation rate constants $k_{d\_app\_early}$ in the early phase (181–211 s) and $k_{d\_app\_late}$ in the late phase (480–780 s) of the lectin nano-block oligomers. The apparent dissociation rate constant k_{d_app} of rACG was calculated using the data in the entire dissociation time (181–780 s).

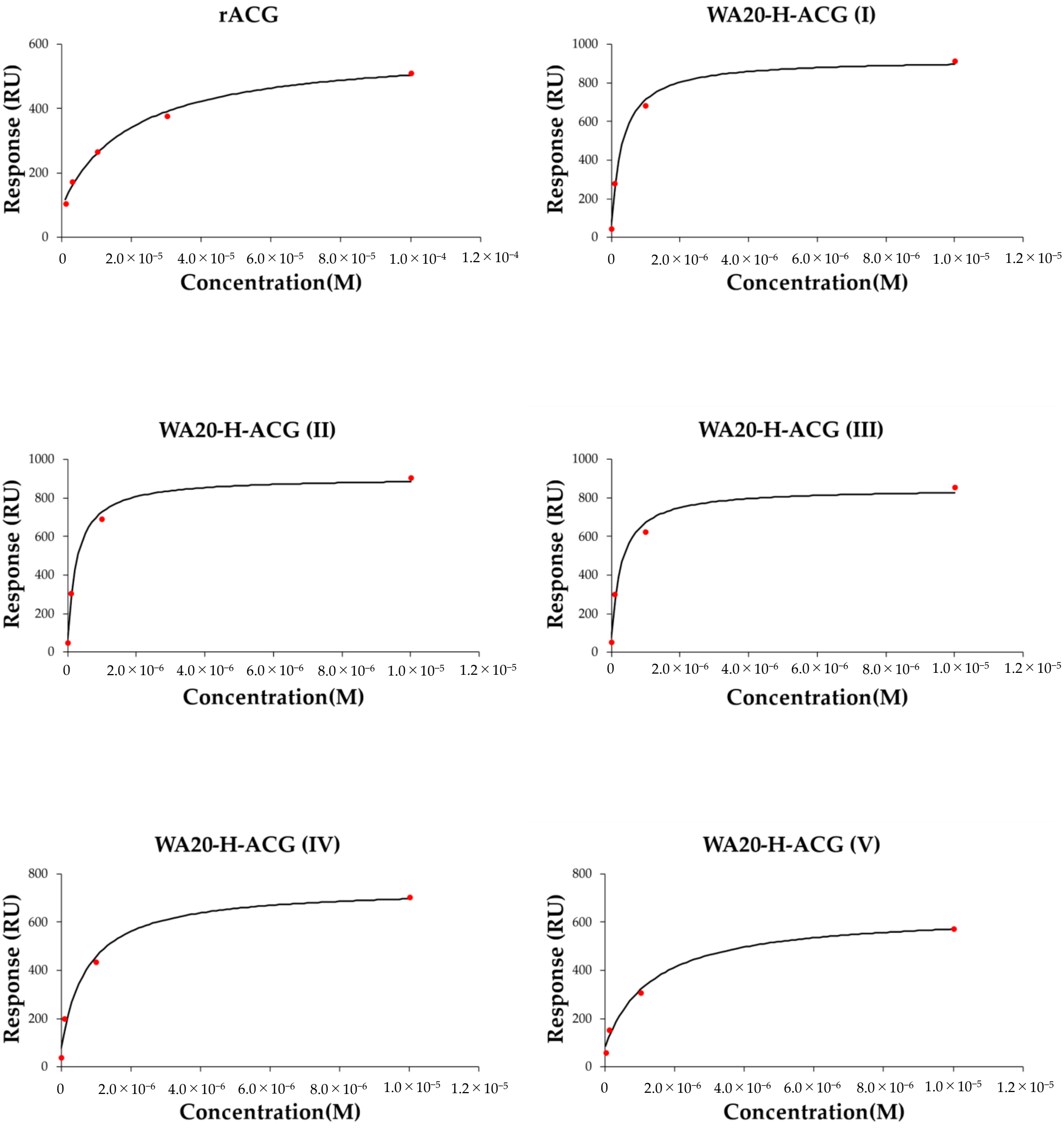

Figure S15. Steady state analysis of SPR data of the WA20-H-ACG oligomers and rACG. The calculation of apparent amount of maximum binding (R_{max_app}) and apparent dissociation constant (K_{D_app}) was performed by approximately fitting the data at several protein concentrations of each sample to the 1:1 binding steady state affinity model in BIAevaluation software (version 3.0) (Cytiva).

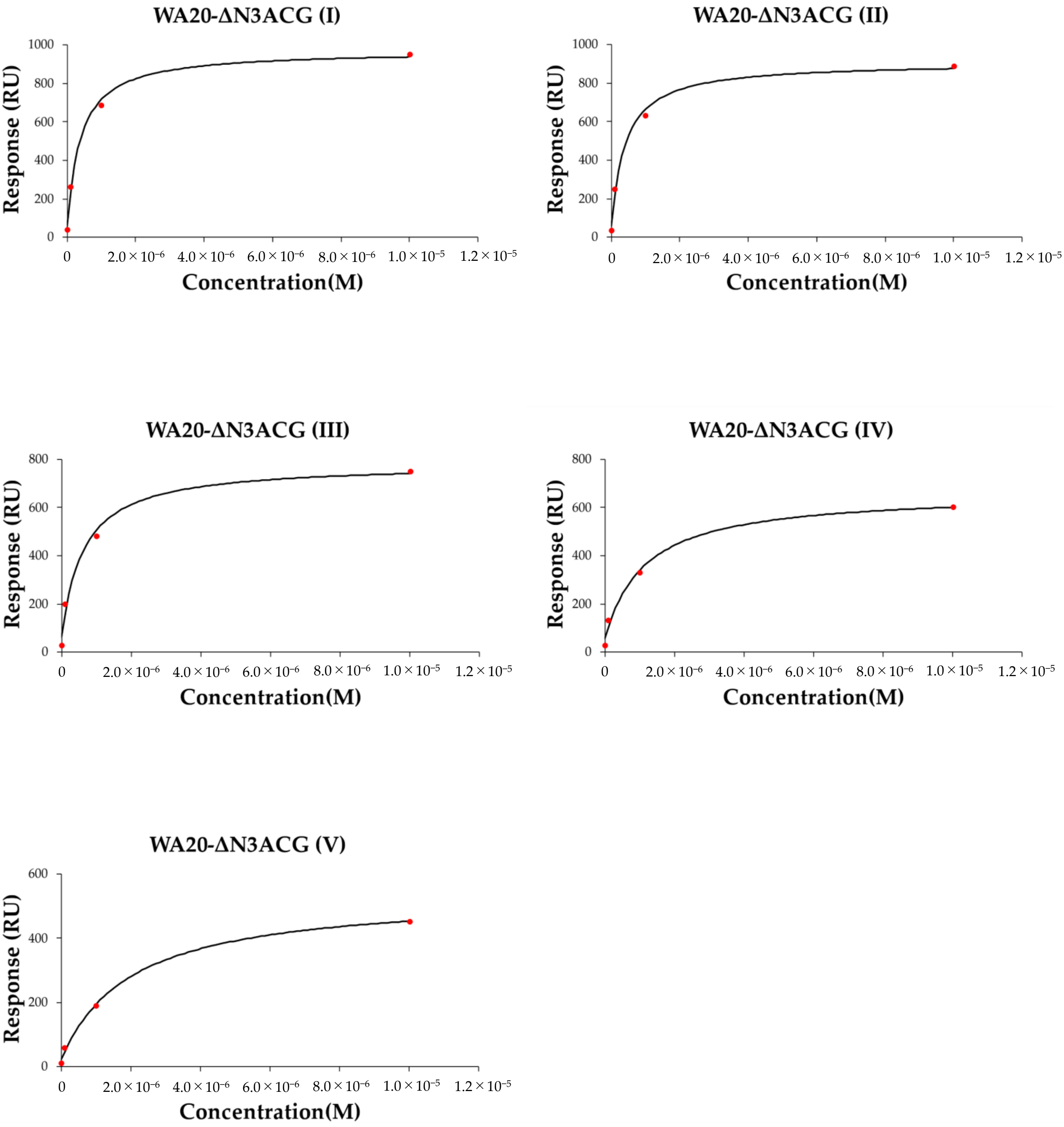

Figure S16. Steady state analysis of SPR data of the WA20-ΔN3ACG oligomers. The calculation of the apparent amount of maximum binding ($R_{\mathrm{max\_app}}$) and apparent dissociation constant ($K_{\mathrm{D\_app}}$) was performed by approximately fitting the data at several protein concentrations of each sample to the 1:1 binding steady state affinity model in BIAevaluation software (version 3.0) (Cytiva).

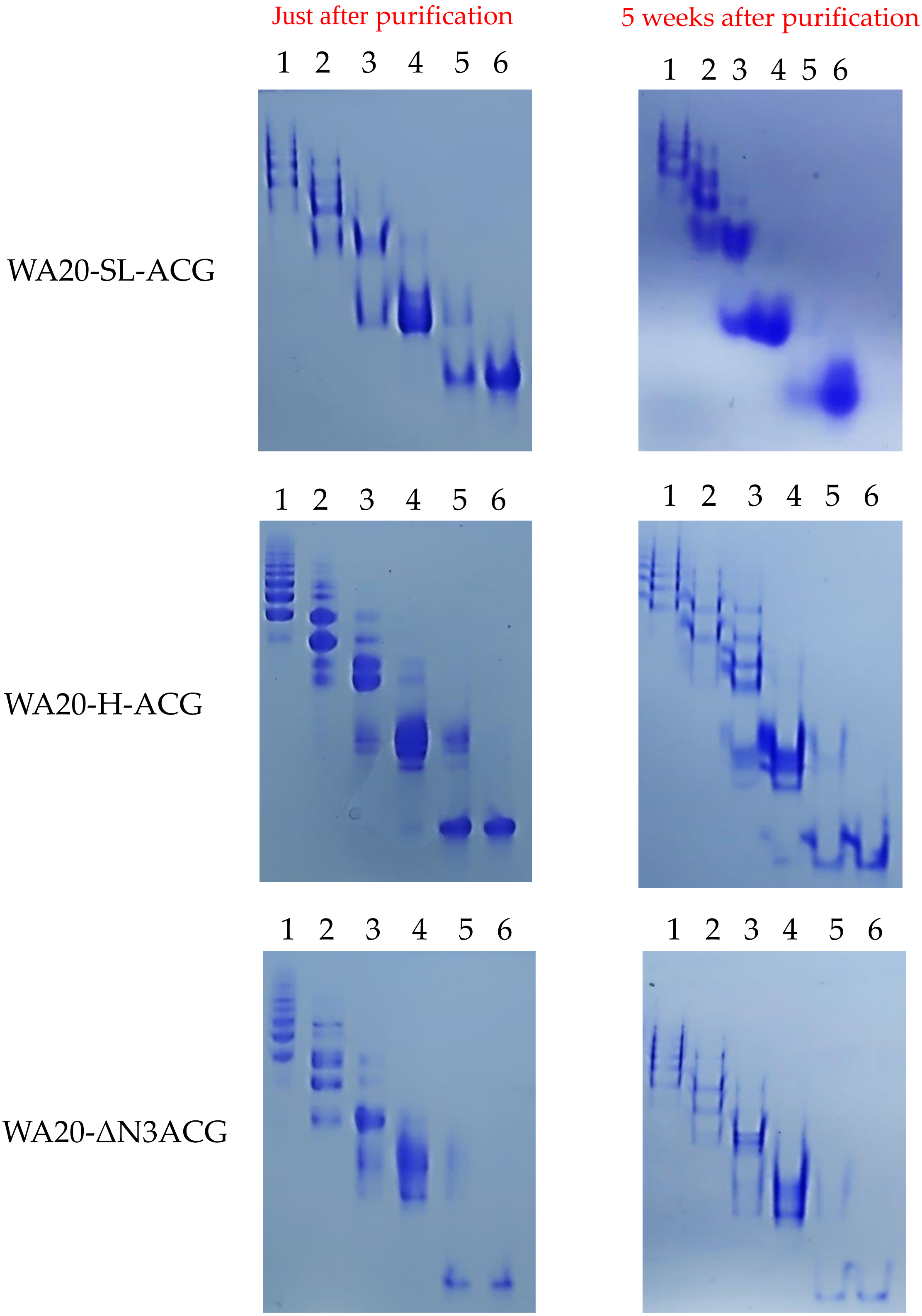

Figure S17. Native PAGE analyses of the lectin nano-block oligomers just after SEC purification and 5 weeks after SEC purification.
Native PAGE of the SEC-fractionated samples of WA20-SL-ACG (top), WA20-H-ACG (middle) and WA20-ΔN3ACG (bottom) with a Superdex 200 increase 10/300 GL column (Cytiva). The fractionated samples were stored at 4 °C for 5 weeks, and then native PAGE of the same samples were performed again.

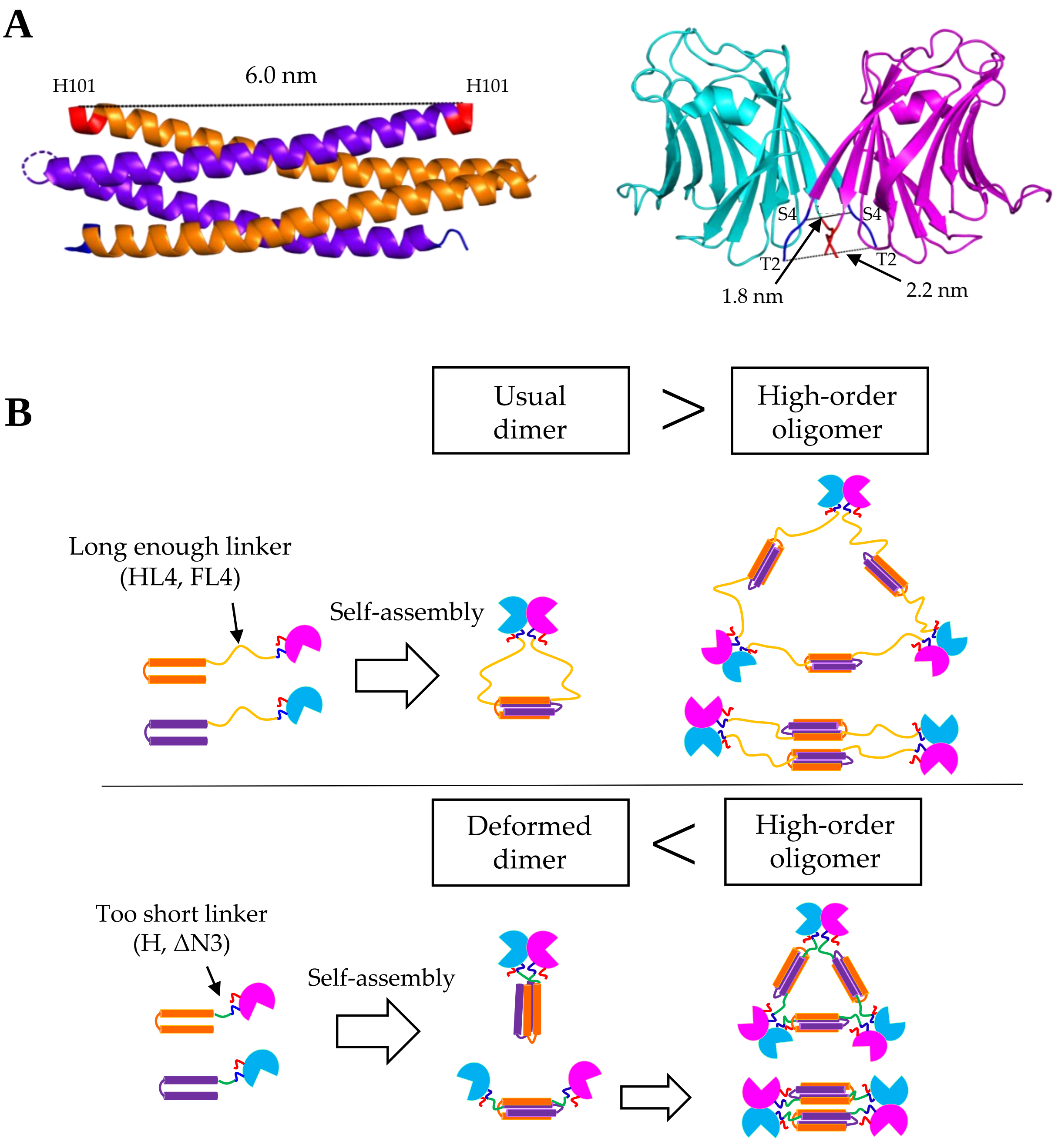

Figure S18. Schematic diagrams of oligomerization of the lectin nano-blocks affected by the linker length.
(**A**) Distance between the C-termini of WA20 (PDB ID: 3VJF) and distance between the N-termini of ACG (PDB ID: 1WW7). (**B**) Schematic models of oligomerization of the lectin nano-blocks affected by the linker length. When the linker of the lectin nano-blocks between WA20 and ACG is long (FL4, HL4), the lectin nano-blocks preferentially form dimer because of the enough length for both domains of WA20 and ACG to form dimers simultaneously. When the linker of the lectin nano-blocks between WA20 and ACG is too short (H, ΔN3), the lectin nano-blocks preferentially form tetramer and higher oligomers because of the too short linker for both domains of WA20 and ACG to form usual dimers simultaneously.

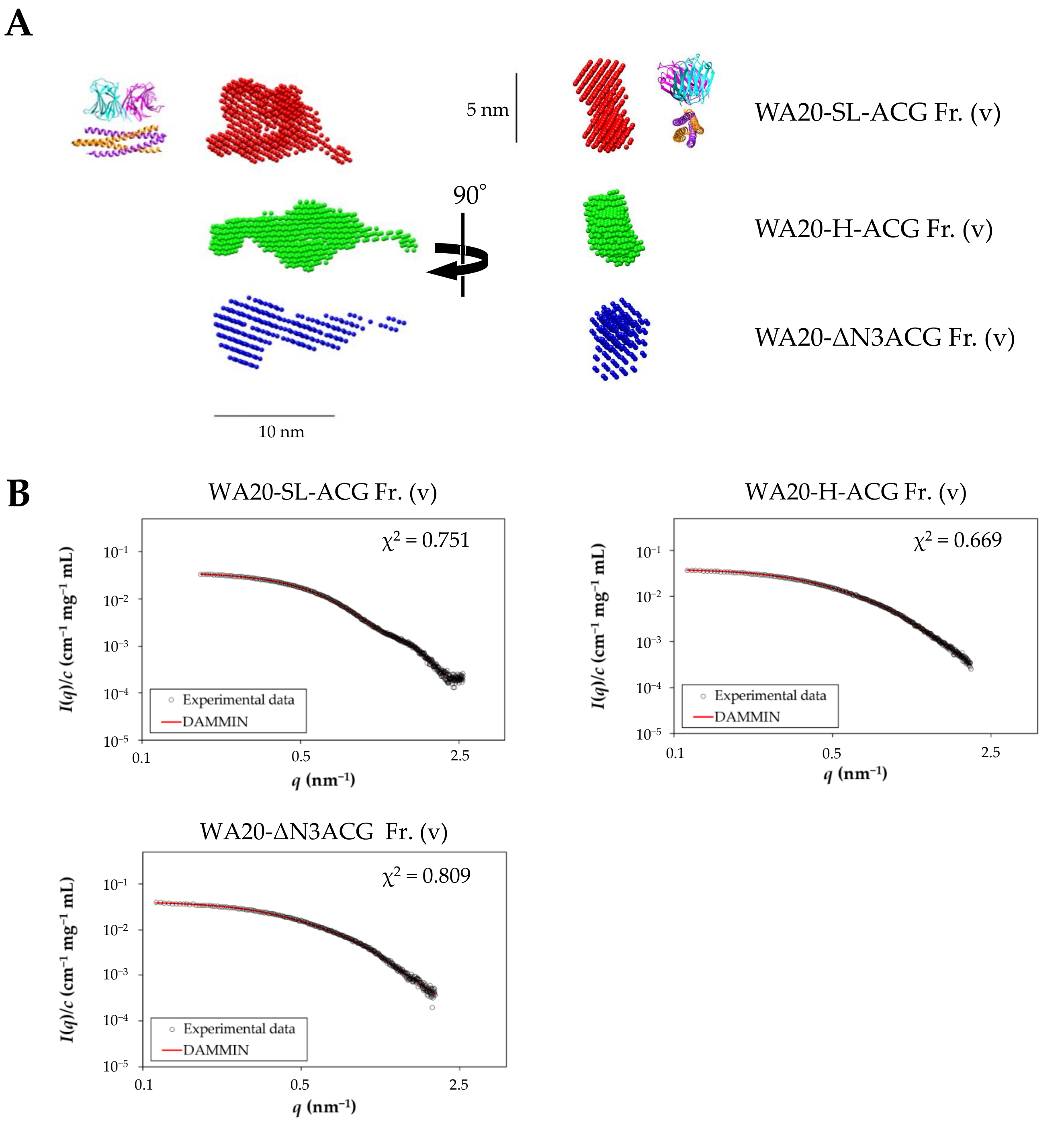

Figure S19. Dummy atom modelling of dimers of the lectin nano-blocks.
(**A**) Dummy atom models of the lectin nano-block dimers of WA20-SL-ACG, WA20-H-ACG, and WA20-ΔN3ACG. The models were constructed based on the SAXS data using the ab initio modelling programs DAMMIF, DAMAVER, and DAMMIN without a symmetry constraint. Ribbon representations of the crystal structures of WA20 (PDB ID: 3VJF) and ACG (PDB ID: 1WW7) are shown as references.
(**B**) Plots of the scattering curves calculated from the DAMMIN models fitting to the experimental SAXS data. The concentration-normalized SAXS intensity $I(q)/c$ of the lectin nano-block dimers (black open circle) and that optimized by the DAMMIN procedure (red line). The χ^2 value represents the degree of fitting between the experimental data and the data calculated from the DAMMIN model.

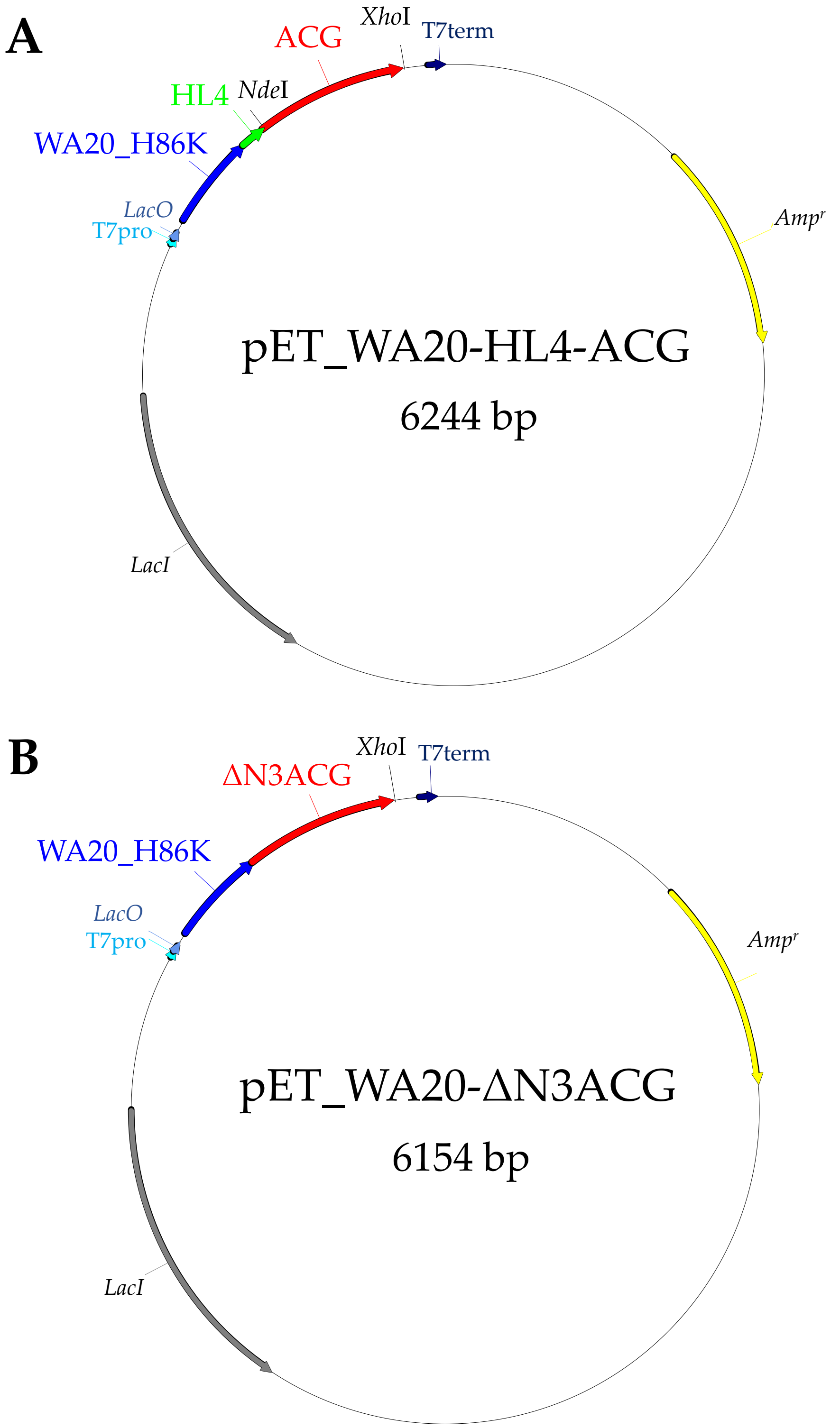

Figure S20. Maps of expression plasmids for the lectin nano-blocks, (**A**) WA20-HL4-ACG and (**B**) WA20-ΔN3ACG.
Expression plasmids of the other lectin nano-blocks were constructed by replacing the HL4 linker (green) of pET_WA20-HL4-ACG with the other linker (FL4, SL, or H).

Table S1. The list of glycans used for glycoconjugate microarray analysis.

Number	Trivial name	Presentation	Glycans
1	αFuc	PAA	Fucα1-PAA
2	Fucα2Gal	PAA	Fucα1-2Galβ1-PAA
3	Fucα3GlcNAc	PAA	Fucα1-3GlcNAcβ1-PAA
4	Fucα4GlcNAc	PAA	Fucα1-4GlcNAcβ1-PAA
5	H type1	PAA	Fucα1-2Galβ1-3GlcNAcβ1-PAA
6	H type2	PAA	Fucα1-2Galβ1-4GlcNAcβ1-PAA
7	H type3	PAA	Fucα1-2Galβ1-3GalNAcα1-PAA
8	A	PAA	GalNAcα1-3(Fucα1-2)Galβ1-4GlcNAcβ1-PAA
9	B	PAA	Galα1-3(Fucα1-2)Galβ1-4GlcNAcβ1-PAA
10	Lea	PAA	Galβ1-3(Fucα1-4)GlcNAcβ1-PAA
11	[3S]Lea	PAA	(3OSO3)Galβ1-3(Fucα1-4)GlcNAcβ1-PAA
12	Leb	PAA	Fucα1-2Galβ1-3(Fucα1-4)GlcNAcβ1-PAA
13	Lex	PAA	Galβ1-4(Fucα1-3)GlcNAcβ1-PAA
14	Ley	PAA	Fucα1-2Galβ1-4(Fucα1-3)GlcNAcβ1-PAA
15	αNeu5Ac	PAA	Neu5Acα2-PAA
16	αNeu5Gc	PAA	Neu5Gcα2-PAA
17	Siα2	PAA	Neu5Acα2-8Neu5Acα2-PAA
18	Sia3	PAA	Neu5Acα2-8Neu5Acα2-8Neu5Acα2-PAA
19	3'SiaLec	PAA	Neu5Acα2-3Galβ1-3GlcNAcβ1-PAA
20	3'SL	PAA	Neu5Acα2-3Galβ1-4Glcβ1-PAA
21	3'SLN	PAA	Neu5Acα2-3Galβ1-4GlcNAcβ1-PAA
22	sLea	PAA	Neu5Acα2-3Galβ1-3(Fucα1-4)GlcNAcβ1-PAA
23	sLex	PAA	Neu5Acα2-3Galβ1-4(Fucα1-3)GlcNAcβ1-PAA
24	6'SL	PAA	Neu5Acα2-6Galβ1-4Glcβ1-PAA
25	FET	Glycoprotein	Fetuin (Complex-type N-glycans and O-glycans)
26	AGP	Glycoprotein	α1-acid glycoprotein (Complex-type N-glycans)
27	TF	Glycoprotein	Transferrin (Complex-type N-glycans)
28	TG	Glycoprotein	Porcine thyroglobulin (Complex and high-mannnose-type N-glycans, and O-glycans)
29	βGal	PAA	Galβ1-PAA
30	[3S]βGal	PAA	(3OSO3)Galβ1-PAA
31	A-di	PAA	GalNAcα1-3Galβ1-PAA
32	Lac	PAA	Galβ1-4Glcβ1-PAA
33	Lec	PAA	Galβ1-3GlcNAcβ1-PAA
34	[3'S]Lec	PAA	(3OSO3)Galβ1-3GlcNAcβ1-PAA
35	LN	PAA	Galβ1-4GlcNAcβ1-PAA
36	[3'S]LN	PAA	(3OSO3)Galβ1-4GlcNAcβ1-PAA
37	[6S]LN	PAA	Galβ1-4(6OSO3)GlcNAcβ1-PAA
38	[6'S]LN	PAA	(6OSO3)Galβ1-4GlcNAcβ1-PAA
39	βGalNAc	PAA	GalNAcβ1-PAA
40	di-GalNAcβ	PAA	GalNAcβ1-3GalNAcβ1-PAA
41	LDN	PAA	GalNAcβ1-4GlcNAcβ1-PAA
42	Gα2	PAA	GalNAcβ1-4Galβ1-4Glcβ1-PAA
43	Asialo-FET	Glycoprotein	Asialo fetuin (Desialylated complex-type N- and O-glycans)
44	Asialo-AGP	Glycoprotein	Asialo α1-acid glycoprotein (Desialylated complex-type N-glycans)
45	Asialo-TF	Glycoprotein	Asialo transferrin (Desialylated complex-type N-glycans)
46	Asialo-TG	Glycoprotein	Asialo porcine thyroglobulin (Desialylated complex-type N-glycans, high-mannose-type N-glycans)
47	βGlcNAc	PAA	GlcNAcβ1-PAA
48	[6S]βGlcNAc	PAA	(6OSO3)GlcNAcβ1-PAA
49	Agalacto-Fet	Glycoprotein	Agalacto fetuin (Agalactosylated complex-type N- and O-glycans)
50	Agalacto-AGP	Glycoprotein	Agalacto α1-acid glycoprotein (Agalactosylated complex-type N- and O-glycans)
51	Agalacto-TF	Glycoprotein	Agalacto transferrin (Agalactosylated complex-type N-glycans, high-mannose-type N-glycans)
52	OVM	Glycoprotein	Ovomucoid (Complex-type N-glycans)
53	OVA	Glycoprotein	Ovoalbumin (Hybrid-type N-glycans)
54	αMan	PAA	Manα1-PAA
55	βMan	PAA	Manβ1-PAA
56	[6P]Man	PAA	(6OPO4)Manα1-PAA
57	INV	Glycoprotein	Yeast invertase (High mannose-type N-glycans)
58	Tn	PAA	GalNAcα1-PAA
59	Core1	PAA	Galβ1-3GalNAcα1-PAA
60	Core2	PAA	Galβ1-3(GlcNAcβ1-6)GalNAcα1-PAA
61	Core3	PAA	GlcNAcβ1-3GalNAcα1-PAA
62	Core4	PAA	GlcNAcβ1-3(GlcNAcβ1-6)GalNAcα1-PAA
63	Forssman disaccharide	PAA	GalNAcα1-3GalNAcβ1-PAA
64	Core6	PAA	GlcNAcβ1-6GalNAcα1-PAA
65	Core8	PAA	Galα1-3GalNAcα1-PAA
66	[3'S]Core1	PAA	(3OSO3)Galβ1-3GalNAcα1-PAA
67	Galβ-Core3	PAA	Galβ1-4GlcNAcβ1-3GalNAcα1-PAA
68	Asialo-BSM	Glycoprotein	Asialo bovine submaxillary mucin (Tn)
69	Asialo-GP	Glycoprotein	Asialo human glycophorin MN (T)
70	STn	PAA	Neu5Acα2-6GalNAcα1-PAA
71	STn (Gc)	PAA	Neu5Gcα2-6GalNAcα1-PAA
72	ST	PAA	Neu5Acα2-3Galβ1-3GalNAcα1-PAA
73	Siaα2-6Core 1	PAA	Galβ1-3(Neu5Acα2-6)GalNAcα1-PAA
74	BSM	Glycoprotein	Bovine submaxillary mucin (Sialyl Tn)
75	GP	Glycoprotein	Human glycophorin (Disialyl T and sialyl Tn)
76	αGal	PAA	Galα1-PAA
77	Galα1-2Gal	PAA	Galα1-2Galβ1-PAA
78	Galα1-3Gal	PAA	Galα1-3Galβ1-PAA
79	Galα1-3Lac	PAA	Galα1-3Galβ1-4Glcβ1-PAA
80	Galα1-3LN	PAA	Galα1-3Galβ1-4GlcNAcβ1-PAA
81	Galα1-4LN	PAA	Galα1-4Galβ1-4GlcNAcβ1-PAA
82	Melibiose	PAA	Galα1-6Glcβ1-PAA
83	αGlc	PAA	Glcα1-PAA
84	βGlc	PAA	Glcβ1-PAA
85	Maltose	PAA	Glcα1-4Glcβ1-PAA
86	-	-	-
87	CSA	BSA	Chondoroitin Sulfate A-BSA
88	CSB	BSA	Chondoroitin Sulfate B-BSA
89	HS	BSA	Heparan Sulfate-BSA
90	HP	BSA	Heparin-BSA
91	KS	BSA	Keratan Sulfate-BSA
92	αRha	PAA	Rhamnoseα1-PAA
93	Mannan (SC)	Glycoprotein	S. cerevisiae mannan
94	Mannan (CA)	Glycoprotein	C.albicans mannan
95	Zymosan	Glycoprotein	Zymosan
96	Chitobiose	PAA	GlcNAcβ1-4GlcNAcβ1-PAA
97	BSA	BSA	-
98	Negative PAA	PAA	-
99	Marker		
100	BG		

Table S2. Oligonucleotide primer sequences used in this study.
Red letters correspond to the codon for an amino acid substitution.

Primer name	Sequence (5′→3′)
T7 terminator primer	GCTAGTTATTGCTCAGCGG
WA20_H86K_Fw	GACACCGTGCATCATTTCAAGAACAAATTGCAGGAGC
WA20_caCatg_Fw	gaaggagatatacacATGTATGGCAAGTTGAACAAG
H_ACG_Nterm_Fw	catATGACCACTTCAGCCGTGAACATCTAC
ACG_Nterm-3_Fw	TCAGCCGTGAACATCTACAACATTAGC
WA20_Cterm_Rv	GCGATGTACAAGGTGGTGGAAG
